# Nondestructive Estimation of Hazelnut (*Corylus avellana* L.) Terminal Velocity and Drag Coefficient Based on Some Fruit Physical Properties Using Machine Learning Algorithms

**DOI:** 10.3390/foods12152879

**Published:** 2023-07-28

**Authors:** Onder Kabas, Mehmet Kayakus, Georgiana Moiceanu

**Affiliations:** 1 Department of Machine, Technical Science Vocational School, Akdeniz University, Antalya 07070, Türkiye; 2 Department of Management Information Systems, Faculty of Social Sciences and Humanities, Akdeniz University, Antalya 07600, Türkiye; mehmetkayakus@akdeniz.edu.tr; 3 Department of Entrepreneurship and Management, Faculty of Entrepreneurship, Business Engineering and Management, University Politehnica of Bucharest, 060042 Bucharest, Romania

**Keywords:** artificial neural network, aerodynamic properties, regression, SVM, MSE

## Abstract

Hazelnut culture originated in Turkey, which has the highest volume and area of hazelnut production in the world. For the design and sizing of equipment and structures in agricultural operations for the hazelnut industry, especially harvesting operations and post-harvest operations, it is essential that an understanding of hazelnuts’ aerodynamic properties, i.e., terminal velocity and drag coefficient, is acquired. In this study, the moisture, mass, density, projected area, surface area, and geometric diameter were used as independent variables in the data set, and the dependent variables terminal velocity and drag coefficient estimation were determined. In this study, logistic regression (LR), support vector regression (SVR), and artificial neural networks (ANNs) were used based on machine learning methods. When the results were evaluated according to R^2^ (determination coefficient), MSE (mean squared error), and MAE (mean absolute error) metrics, it was seen that the most successful models were the ANN, SVR, and LR, respectively. According to the R^2^ metric, the ANN method achieved 91.5% for the terminal velocity of hazelnuts and 85.9% for the drag coefficient of hazelnuts. Using the independent variables in the study, it was seen that the terminal velocity and drag coefficient value of hazelnuts could be successfully estimated.

## 1. Introduction

Turkey is the homeland of hazelnut culture. Hazelnuts, which are the nut of the hazel tree, are also called cobnuts and filbert nuts, depending on the species. There is a smooth shell surrounding a fibrous husk covering the cob, which is generally round or oval and about 15–25 mm (0.59–0.98 in) in length and 10–15 mm (0.39–0.59 in) in diameter. Globally, hazelnuts come in second place to almonds as far as cultivation is concerned. They have an important place in human nutrition and are mainly used in confectionery, but they are also used as table nuts and in oil production [1].

According to data from the Food and Agriculture Organization, the world hazelnut production area is 966,196 hectares; approximately 75.4% of hazelnut production is in Turkey, about 8.1% is in Italy, 4.0% is in Azerbaijan, 1.9% is in Turkey, and 1.8% is in the USA. The hazelnut is therefore a valuable economic commodity. Turkey is the largest hazelnut producer in the world, with 69% of the global hazelnut production (about 776 000 tons out of the total world production of about 1,125,000 tons) [2]. Additionally, Turkey is the largest exporter of hazelnuts, conducting about 80% of the world’s hazelnut exports [3].

Considering the importance of hazelnuts for Turkey, the systems used in the process—from harvest to reaching the consumer—should be designed in an optimum way to increase work efficiency, save time, and prevent labor losses. In this chain, the aerodynamic properties of the hazelnut for the optimum design of the machinery and systems used for the classification, drying, transportation, and cleaning of the hazelnuts under suitable conditions are important factors to know. Additionally, in order to harvest hazelnuts, perhaps using a stationary thresher and cleaning unit, the physical and aerodynamic properties of hazelnuts are also important to know [4].

For the optimization of the systems used in hazelnut processing, the aerodynamic properties such as the terminal velocity and drag coefficient are important factors to consider. These properties (the terminal velocity and drag coefficient) are affected by numerous factors such as the humidity, mass, density, projected area, surface area, and geometric diameter [5].

Aerodynamic tests with different agricultural products have determined terminal velocity as a function of moisture content. However, some studies showed that the terminal velocity of an agricultural product also changes according to the mass, geometric diameter (form), volume, density, and superficial projected area of the product [4,6,7]. In addition, in a study on the separation of both damaged and undamaged seeds, the drag coefficient was determined as a function of the mean geometric diameter [8].

The terminal velocity and drag coefficient can be calculated or measured in the laboratory according to the specified fruit characteristics (humidity, mass, density, projected area, surface area, and geometric diameter). Two commonly used methods for the experimental measurement of terminal velocity are the drop and suspension methods [9]. Both methods involve a very long, laborious process, and they are also very time consuming and labor intensive. In current times, when energy and labor are crucial and time is of the essence, nontraditional methods can be used, instead of experimental methods, to accurately predict these desired properties.

It is frequently challenging to establish the relationship between the dependent variables and independent variables in system/process phenomena [10]. It is challenging to map the behavior of a system or process with a mathematical model in such circumstances, and even when answers are established, such mathematical models are frequently complicated, nonlinear, and parallel. The physical characteristics of hazelnut fruits and their aerodynamic characteristics do not have a known numerical relationship. As a result, machine learning processes offer a more effective method of solving this kind of problem [11]. In recent years, there has been an increase in the usage of some machine learning technologies to solve these problems. Using these techniques, solution-oriented approaches can be achieved via fast and simple simulations [12]. Such effective methods are required for the precise representation and identification of descriptive parameters utilized in agricultural and food product quality evaluation. In an input–output coupling, machine learning provides nonlinear models that can forecast current and future values [13]. The physical, mechanical, and qualitative aspects of fruit have been studied using various network architectures of artificial neural networks, including different inputs, network structures, training algorithms, iteration counts, and so on. Suitable ANN models for predicting physical and mechanical qualities have been identified by combining different combinations. The ANN method was applied to estimate the typical physiological changes in pears, and the ANN model produced the best estimation based on real data [14]. An artificial neural network can be used to better estimate the volume and surface area of a fruit according to Ziaratban et al. [15]. Lu et al. [16] used single-hidden-layer ANNs in their asparagus investigation, and the number of neurons in the hidden layer was determined via a trial-and-error method.

Machine learning, which can be defined as a type of application in which computer programs can learn patterns through training data and algorithms, is a sub-branch of artificial intelligence. Its application, which imitates human movements, aims to learn through experience without programming. The learning system of the machine learning algorithm is divided into three main parts. The decision process is used by machine learning algorithms to make a prediction or classification. Based on some input data, which may be labeled or unlabeled, an algorithm will generate a prediction about a pattern in the data. An error process is used to evaluate the prediction of the model. If there are known examples, an error function can perform a benchmark to evaluate the accuracy of the model. In the optimization process, if the model fits the data points in the training set better, then the weights are adjusted to reduce the discrepancy between the known sample and the model prediction. By repeating this evaluation and optimizing the process, the algorithm autonomously updates the weights until an accuracy threshold is reached [17].

This study presents an accurate estimation of aerodynamic properties such as terminal velocity and drag coefficient depending on some fruit properties, such as humidity, mass, density, projection area, surface area, and geometric diameter, using a machine learning method. By using machine learning models, we aim to determine the most accurate model given different inputs and network structures. The results obtained can be considered a useful tool when the drying, harvesting, sorting, transportation, separation, trashing, and processing of hazelnuts is developed and optimized.

## 2. Materials and Methods

In this study, hazelnut samples randomly selected from the hazelnut variety (Palaz) were used in all trials. Experiments were carried out in 4 replicates using 30 hazelnuts in each replicate. Samples were obtained from different hazelnut growers for the 2022 harvest season in Samsun, Turkey. The experiments were carried out as soon as possible after the hazelnuts were purchased. Samples were kept in a +4 C refrigerator for one day until analysis. Experiments were carried out in Akdeniz University Technical Sciences Vocational School Laboratories.

### 2.1. Data Set

The moisture content of the hazelnut variety (Palaz) was determined using the ASAE standard method and found to vary between 3.75 and 20.33% db (db = dry basis) [18]. In order to determine the average linear dimensions (thickness (T), width (W), and length (L)) and mass of the hazelnuts used in the experiments, 30 hazelnut samples were randomly selected for each replication and the dimensions of each hazelnut were measured with a digital caliper with an accuracy of 0.01 mm. The mass (M) was measured using a digital balance with an accuracy of 0.001 g.

**Figure 1:**
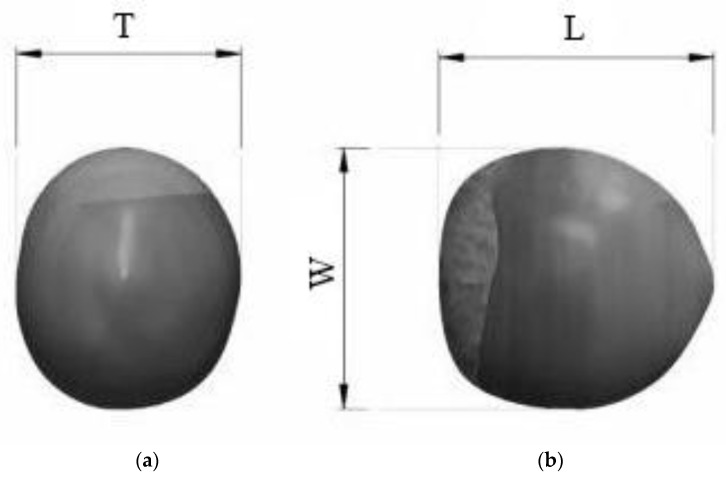
Three dimensions of a hazelnut ((**a**) vertical axis, (**b**) horizontal axis).

The surface area and geometric mean diameter of the hazelnuts were calculated using the following equations [6]:

(1) $$D_{g}={\left(L\times W\times T\right)}^{1/3}$$

(2) $$S=\pi \times D_{g}^{2}$$

where Dg is the geometric mean diameter in mm, L is the length (mm), W is the width (mm), T is the thickness (mm), and S is surface area in mm^2^ (Figure 1).

The projection area (P) was calculated by comparing the projection area with the reference area using Sigma Scan Pro 5 software on hazelnut images taken with a digital camera (Nikon D 5600), as shown in Figure 1b [19].

The hazelnut density (ρ) was measured by the liquid displacement method. Toluene (C7 H8) was used, rather than water, because it was not absorbed by the nuts [20,21].

**Figure 2:**
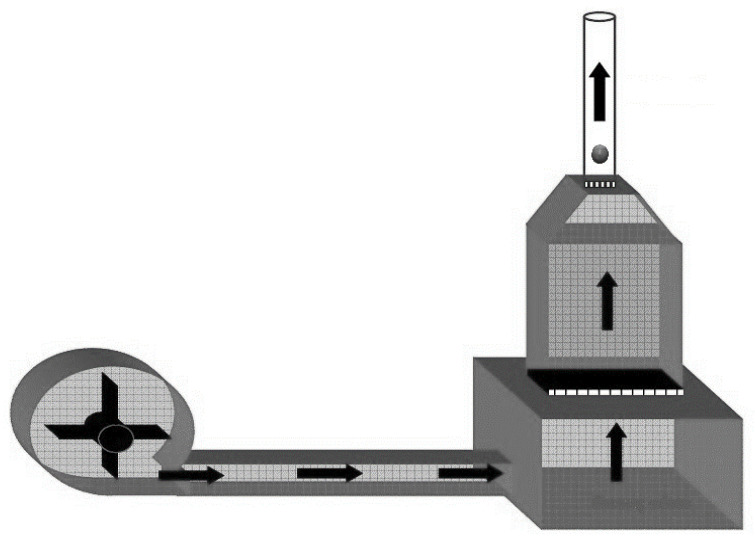
Wind tunnel.

The terminal velocity was determined using a wind tunnel (Figure 2). A radial fan driven by a 1.5 kW electric motor was used to create the airflow in the wind tunnel. The air volume was adjusted by means of a regulating valve placed at the air inlet opening. The air, created by a radial fan, was delivered to the pressure chamber. The pressure chamber ensures that the incoming air flow was close the laminar flow. Between the pressure chamber and the air outlet duct, to which the test duct is connected, a honeycomb-shaped piece, called a rectifier, was placed to ensure a near laminar flow. A test channel made of a transparent material was placed in the air outlet duct to study the movement of the test material in the air. For each test, a sample (nut) was dropped into the test channel from the top of the wind tunnel and the air velocity at the moment the sample was suspended in the air was measured with a digital hot-wire anemometer with an accuracy of 0.1 ms^−1^ [22].

Furthermore, if the terminal velocity of the sample is known, the drag coefficient C_d_ can be calculated by [6,23]:

(3) Cd=2mg(ρp−ρf)ρpρfApVt2

where C_d_ is the drag coefficient (−), g is the acceleration due to gravity (9.81 ms−^2^), m is the mass of samples (kg), ρ_p_ is the density of samples (kgm^−3^), ρ_f_ is the density of air (1.206 kgm^−3^), Ap is the projected area of the particle (m^2^), and V_t_ is the terminal velocity (ms^−1^).

### 2.2. Machine Learning Methods

Machine learning is the modelling of systems with computers that make predictions by making inferences on data with mathematical and statistical operations. Machine learning is a branch of artificial intelligence used to classify and forecast data by mimicking the way humans learn. Machine learning is used in many different fields, such as medicine, the automotive industry, marketing, and speech recognition technologies. For example, in natural language processing, it is used in tasks such as analyzing and understanding texts, translation, or detecting emotional meaning. In image processing, it is effective in tasks such as recognizing objects from images, face recognition, or image classification [24,25].

In this study, artificial neural network, logistic regression, and support vector regression methods were used as machine learning methods. The decision to use these methods was influenced by the characteristics of the models, the suitability of the data set, and the studies in the specialty literature. The facts that these models produced successful and fast results, according to the size and characteristics of the data set, and the lack of these studies in the literature for originality were reasons for the selection of these models.

#### 2.2.1. Logistic Regression

Logistic regression is a supervised machine learning method used to linearly model the relationship between dependent and independent variables. It is a type of statistical analysis used to predict the outcome of a dependent variable based on previous observations. Logistic regression can be used if the type of variable to be predicted is categorical. The major difference between logistic regression and linear regression is how it applies the line to separate the two classes. While linear regression uses least squares to draw the optimal line, logistic regression uses maximum likelihood [26].

The aim of logistic regression is to determine the effect of one or more independent variables on the dependent variable when the dependent variable is qualitative. Logistic regression analysis is a method that calculates the estimated values of the dependent variable as a probability and allows estimation in accordance with the probability rules. In logistic regression analyses, the ratio of the probability of an event occurring and the probability that the event will not occur is called the odds ratio. p(x) represents the probability of an event occurring and 1 − p(x) represents the probability that the event will not occur [27,28]. The odds formula is shown in the following:

(4) $$Odds=\frac{p(x)}{1-p(x)}$$

The odds take values between 0 and +∞. The odds ratio is asymmetrical. It is transformed into a symmetrical ratio by taking its natural logarithm. By taking the natural logarithm of both sides of the logistic function, which can be used in linear regression analysis through this ratio, a linear structure is obtained [29].

(5) $$\ln\left(Odds\right)=ln\frac{P}{1-P}$$

While the odds of a probability take values between 0 and +∞, the logit value of the same probability takes values between −∞ and + ∞ [27].

- As P increases, logit(P) also increases.
- If P < 0.5, logit(P) is negative.
- If P > 0.5, the logit(P) takes positive values.
- When P is between 0 and 1, logit(P) can take values in the line of real numbers [30].

#### 2.2.2. Artificial Neural Network

Artificial neural networks are a method designed to simulate the way the simple biological nervous system works. Simulated nerve cells contain neurons, and these neurons connect to each other in various ways to form a network. These networks have the capacity to memorize and reveal the relationship between data sets [31].

Artificial nerve cells have five basic elements. Each artificial nerve cell has inputs that receive external information, weights that process incoming information and create connections, a summation function, an activation function, and output elements that present the processed information to the outside. The inputs represent information from the outside. The summation function calculates the net input entering the cell. According to the ANN model to be applied, various functions can be used. Generally, the addition function is the summation of the information coming into the cell by multiplying the weights of that information [32]. Equation (6) shows the net input.

(6) $$NET=\sum_{i}^{N}X_{i}W_{i}$$

where X is the inputs, W is the weight value and n represents the total number of inputs that have entered a cell.

The activation function establishes a connection between input and output. It processes the information from the collection function and creates the output information. This function, like the summation function, has various functions according to the ANN model to be implemented. The most used activation function is Sigmoid [33]. Equation (7) shows the Sigmoid function.

(7) $$f\left(NET\right)=\frac{1}{1+e^{-NET}}$$

The output contains the values produced by the activation function.

Artificial neural networks are divided into four groups: single-layer and multi-layer perceptrons, feed-forward networks, and feedback networks. Single-layer networks consist of inputs and an output. They can have multiple input values. In a single-layer network, the output function is linear and takes a value of 1 or −1. Multi-layered ANNs consist of input, hidden, and output layers. In feedforward ANNs, neurons move regularly from input to output. Only one layer connects with the next layer. The information in the input is transmitted to the neurons in the hidden layer without any change. It is then processed at the output layer and transferred to the output. In a feedback ANN, unlike forward-feed networks, the output of a neuron is not given only as an input to the layer of neurons that follows it. It can connect to the previous layer or to a neuron located in its own layer [34,35].

#### 2.2.3. Support Vector Regression

Support vector machine (SVM) is a supervised machine learning algorithm that can be used for classification or regression problems. SVM is suitable for linear or non-linear classification and regression problems. SVM basically separates data from multiple classes in the most appropriate way. For this purpose, it draws lines called decision boundaries, or in other words, a hyperplane to separate the points in a plane. This line is intended to be at the maximum distance for the points of both classes. Support vector machine was first proposed by Vapnik [36], inspired by the statistical learning method. This method was developed for classification operations. Then, the support vector regression (SVR) method was developed to solve prediction problems [37]. When SVR is applied, it ensures that the drawn range encapsulates the maximum point (Figure S5).

SVR is a regression model that allows us to define how many errors can be accepted in the generated model. Based on the errors entered, it finds a suitable line or creates a hyperplane. Therefore, the SVR method is applied in an attempt to minimize the prediction error and, in this way, aims to find a function that approaches the training data (Figure S6). The flatness of the function is maximized, reducing the risk of being stuck in local values [38,39].

### 2.3. Data Preprocessing

**Figure 3:**
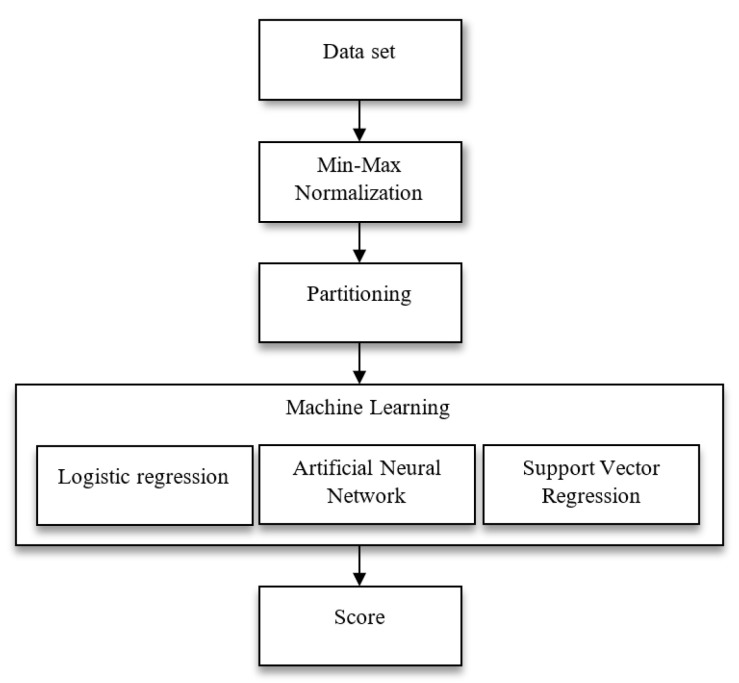
The flow diagram of the system.

In this study, hazelnut terminal velocity and drag coefficient estimations were performed using three different machine learning methods. The flow diagram of the system is shown in Figure 3.

**Figure 4:**
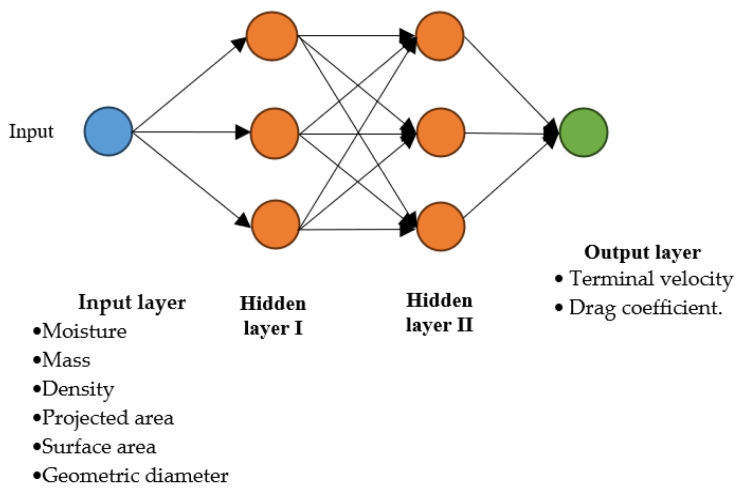
ANN Model.

In this study, the moisture, mass, density, projected area, surface area, and geometric diameter were used as independent variables in the data set, and the dependent variables of terminal velocity and drag coefficient were predicted. There are 30 data samples for each independent variable in the data set. Figure 4 shows the ANN model designed for the study.

Normalization is a statistical method used to express values in different value ranges in the data set in the same range. The normalization process reduces the training time and significantly increases the performance of machine learning methods [40]. Min-max, median, sigmoid, decimal scaling, and z-score methods are the most frequently used normalization techniques in the literature. In this study, the data samples were normalized between 0 and 1 using the min-max method.

The next stage of the study is partitioning. While training the models, the whole data set is divided into two parts: a training and a test set. The data used to set the parameters of the models are called training data and the data used to measure the accuracy of the selected model are called test data. In the test data, predictions and real data are compared. Thus, the performance of the model is measured. This partitioning ratio can take different values according to the characteristics of the model. These ratios are generally 60–40%, 70–30%, or 80–20% [41]. In the study, experiments were carried out on the models to divide the data set into training and test sets. As a result of these trials, it was decided to separate the training and test data set as 70–30%.

In the study, the determination coefficient (R^2^), mean squared error (MSE), and mean absolute error (MAE) metrics were used to evaluate and compare the machine learning methods used. The equations of these statistical methods are given below.

(8) $$R^{2}=1-\frac{\mathrm{Unexplained}\;\mathrm{Variation}}{\mathrm{Total}\;\mathrm{Variation}}$$

(9) $$MSE=\frac{1}{n}\sum_{i=1}^{n}e_{i}^{2}$$

(10) $$MAE=\frac{1}{n}\sum_{i=1}^{n}\left|e_{i}\right|$$

where n is the amount of data and e is the error value.

R^2^ is a statistical criterion that we used to evaluate the performance of the model in regression analysis. The great feature of this criterion is that it is extremely convenient for comparing different regression models. R^2^ indicates the power of the equation obtained in the regression analysis to measure the dependent variable. The coefficient of R^2^ is from 0 to 1. The closer its value is to 1, the greater the adaptation of the model to the variable we are trying to explain. The closer it is to zero, the less tight the model will be and therefore the less reliable it will be. A high R^2^ indicates a good regression model fit. The MSE measures the performance of the prediction model. The MSE indicates how close a regression curve is to a series of points [42,43]. Since the MSE takes the squares of the errors, it produces large numerical results when there is a large deviation. In these cases, the MAE can be used instead of MSE. The MAE shows how close a regression curve is to a series of points. The MAE is the average horizontal and vertical distance between each real value and the line that best fits the data. The MAE value can range from 0 to ∞. Estimators with a lower MAE indicate a better performance.

## 3. Results and Discussions

### 3.1. Logistic Regression Analysis

The maximum likelihood method was used to estimate the parameters for the logistic regression model. In this study, the log-likelihood value was −16.449, as a result of 100 iterations. The evaluation of the independent variables used to estimate the terminal velocity of hazelnuts using logistic regression is shown in Table 1.

According to the statistical metric in Table 1, R^2^ varies between 59.6% and 74.9%. These values were close to the acceptable range, and the lowest R^2^ was for surface area and the highest R^2^ was for the moisture variable in estimating the hazelnut terminal velocity. The lowest MSE was 0.023 and the highest was 0.04, and the value was close to the desired value. The MAE ranged from 0.127 to 0.194 and these values were close to the ideal values. According to these results, it was observed that logistic regression showed a close-to-success and close-to-ideal result in estimating the terminal velocity of hazelnuts.

The evaluation of the independent variables used in the estimation of the drag coefficient of hazelnuts using logistic regression is shown in Table 2.

According to the statistical metrics in Table 2, R^2^ varied between 66.4% and 86.3%. These values were close to the acceptable range, and the lowest R^2^ was for density and the geometric diameter variable had the highest R^2^ in estimating the hazelnut drag coefficient. The lowest MSE was 0.012 and the highest was 0.046, and the value was close to the ideal value. The MAE ranged from 0.081 to 0.159 and was close to the ideal values. According to the average results in Table 2, logistic regression showed close to acceptable results in estimating the drag coefficient of hazelnuts.

### 3.2. Artificial Neural Network Analysis

An artificial neural network (ANN) method is a supervised and feedback model. In the process of creating an ANN model, it is necessary to determine the number of hidden layers and the number of neurons in each layer. Although there is no rule for this, the most used method is the trial-and-error method. The number of hidden layers and the number of neurons are the most important parameters that affect the success of the model. In this study, the most successful results were obtained in the model with two hidden layers and three neurons in each layer. For this model, 100 iterations were performed.

The evaluation of the independent variables used to predict the terminal velocity of hazelnuts using artificial neural networks is shown in Table 3.

According to the statistical data in Table 3, R^2^ varied between 88.2% and 94.9%. These values were in the acceptable range, and the lowest R^2^ was for density and the highest R^2^ was for the geometric diameter variable in estimating the terminal velocity of hazelnuts. The lowest MSE was 0.005 and the highest was 0.013, and the value was very close to the desired value. The MAE ranged from 0.053 to 0.083 and they were very close to the ideal values. According to the average results in Table 3, the artificial neural network showed a successful result in estimating the value of the hazelnut terminal velocity. 

The independent variable evaluation used to predict the drag coefficient of hazelnuts using artificial neural networks is shown in Table 4.

According to the statistical data in Table 4, R^2^ varied between 78.5% and 93.2%. These values were in the acceptable range, and in estimating the drag coefficient of hazelnuts, the lowest R^2^ was for moisture and the highest R^2^ was for the density variable. The lowest MSE was 0.007 and the highest was 0.017, and the value was very close to the desired value. The MAE ranged from 0.066 to 0.109 and was very close to the ideal values. According to the average results in Table 4, the artificial neural network showed an acceptable result in estimating the drag coefficient of hazelnuts.

### 3.3. Support Vector Regression Analysis

The other method used in the study was non-linear support vector regression (SVR). The most important parameter affecting the success of the model in SVR is the choice of core function. As a result of tests, it was decided to use the radial basis function (RBF) in the model. The overlapping penalty value in the model was 20 and sigma was set to 0.1. The evaluation of the independent variables used to predict the terminal velocity of hazelnut using SVR is shown in Table 5.

According to the statistical data in Table 5, R^2^ varied between 80.2% and 91.4%. These values were in the acceptable range, and the lowest R^2^ was for density and the highest R^2^ was for the geometric diameter variable in estimating the terminal velocity of hazelnuts. The lowest MSE was 0.009 and the highest was 0.140, and the values were very close to the desired values. The MAE ranged from 0.084 to 0.121 and was very close to the ideal value. According to the average results in Table 5 and Figure S1, the artificial neural network showed an acceptable result in estimating the value of hazelnut terminal velocity.

The evaluation of the independent variables used in the estimation of hazelnut drag coefficient using support vector regression is shown in Table 6.

According to the statistical data in Table 6, R^2^ varied between 65.7% and 91.4%. The values in the table varied, and in estimating the drag coefficient of hazelnut, the lowest R^2^ was for moisture and the highest R^2^ was for density (Figure S2). The lowest MSE was 0.009 and the highest was 0.026, and the value was very close to the desired value. The MAE ranged from 0.090 to 0.136 and was very close to the ideal value. According to the average results in Table 3, the artificial neural network shows a result close to acceptable values in estimating the drag coefficient of hazelnuts.

### 3.4. Discussion

In this study, modelling was performed according to three different machine learning methods: logistic regression (LR), artificial neural networks (ANN) (Figure S3), and support vector regression (SVR). A comparison of the machine learning methods used in the estimation of hazelnut terminal velocity and drag coefficient is shown in Table 7.

An R^2^ of 1 indicates that the test data fit to a linear curve. The R^2^ in Table 7 was 69% for LR, 91.5% for ANN, and 85.9% for SVR. These results were found to be very close to the ideal value. When the MSE is close to zero, the models show better and fewer errors. Therefore, it is desirable that the MSE of the models used in the study is close to zero. In the study, the MSE was 0.031 for LR, 0.009 for ANN, and 0.035 for SVR, which is close to the ideal value. An MAE close to zero indicates a less erroneous prediction. In the study, the MAE value was 0.158 for LR, 0.070 for ANN, and 0.104 for SVR, which is close to the ideal value. According to these results, artificial neural networks, support vector regression, and logistic regression were found to be more successful and have less error than the machine learning methods used to predict hazelnut terminal velocity.

A comparison of the machine learning methods used to predict the drag coefficient of hazelnuts is shown in Table 8.

The R^2^ in Table 8 was 75.6% for LR, 85.9% for ANN, and 80.2% for SVR. According to these results, R^2^ is in the acceptable value range. It was seen that the MSE was 0.027 for LR, 0.012 for ANN, and 0.018 for SVR, which is close to the ideal value. It was seen that the MAE was 0.124 for LR, 0.086 for ANN, and 0.110 for SVR, which is close to the ideal value. According to these results, artificial neural networks, support vector regression, and logistic regression were found to be more successful and less error prone than the machine learning methods used to predict the drag coefficient of hazelnuts (Figure S4).

**Figure 5:**
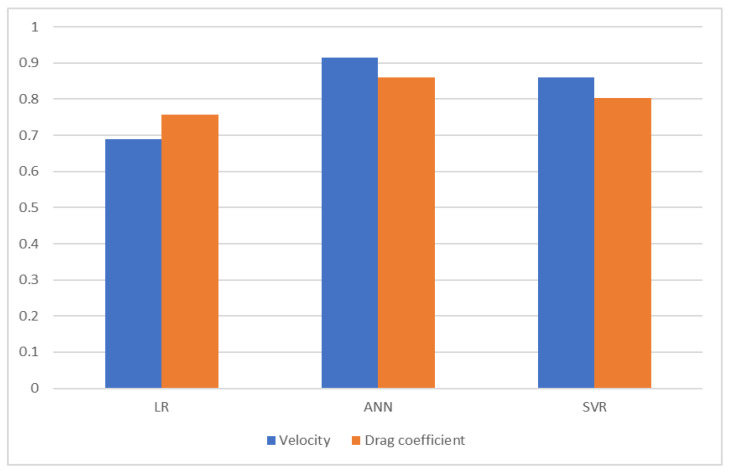
R^2^ of estimation methods.

Figure 5 shows the results of all three models according to the R^2^ metric for estimating the terminal velocity and drag coefficient of hazelnuts. As shown in the figure, the models achieved acceptable values in predicting both dependent variables. In particular, the ANN method is more successful than the others.

**Figure 6:**
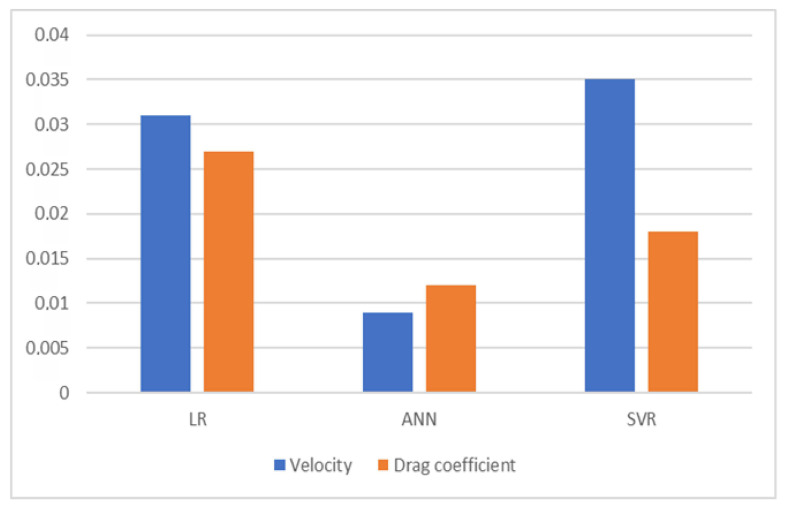
MSE of estimation methods.

Figure 6 shows the results of all three models according to the MSE metric for estimating the terminal velocity and drag coefficient of hazelnuts.

**Figure 7:**
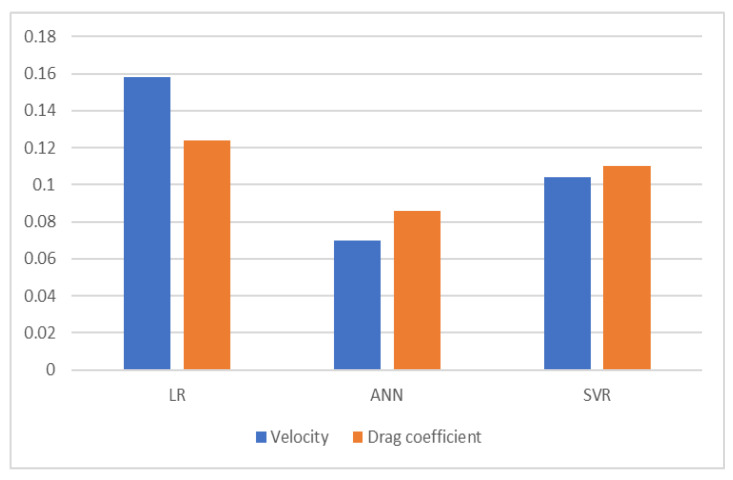
MAE of estimation methods.

Figure 7 shows the results of all three models according to the MAE metric for estimating the terminal velocity and drag coefficient of hazelnut.

## 4. Conclusions

The aerodynamic properties of hazelnuts (terminal velocity and drag coefficient) are major parameters for harvesting and post-harvesting operations. These parameters are very important for the design and modification of machines used in many processes, such as harvesting, drying, transportation, classification, cleaning, etc. In order to determine these properties, measurements with a large number of samples are required. These types of measurements are time consuming, costly, and labor intensive. Additionally, they introduce several measurement errors. Identifying such features with machine learning systems leads to more datasets, attributes, and algorithms for further study, as well as faster and more reliable results for industrial applications such as discrimination, sorting, and prediction processes.

In this study, the terminal velocity and drag coefficient of hazelnuts were successfully predicted by models created using six different independent variables in three machine learning methods. Considering the R^2^ metric in the evaluation of the methods, it was seen that these models can be used to predict the terminal velocity and hazelnut drag coefficient using six independent variables. An MSE and MAE of zero indicate that the models are error free. Since the MSE and MAE metrics are close to zero in this study, it can be concluded that the findings of the study are acceptable and the error rate is low. As a result of the evaluation of the models, it was seen that the most successful methods with the lowest error were artificial neural networks, support vector regression, and logistic regression, respectively.

Machine learning can be used in the food processing industry in a highly practical, fast, and reliable way. In addition, it can help in the determination of physical and engineering properties of agricultural products and in quality assessment industries. The current models and findings will provide important contributions to researchers and designers.

In future studies, the prediction success can be increased by increasing the size of the data sets and adding independent variables to the models. In addition, the effect of using different machine learning methods such as deep learning on the accuracy of the method can be analyzed.

## Figures and Tables

**Table 1:** Evaluation of logistic regression model (the terminal velocity of hazelnuts).

	R^2^	MSE	MAE
Moisture	0.749	0.023	0.127
Mass	0.746	0.023	0.143
Density	0.663	0.037	0.161
Projected area	0.708	0.029	0.159
Surface area	0.596	0.040	0.194
Geometric diameter	0.678	0.032	0.164
Average	0.690	0.031	0.158

**Table 2:** Evaluation of logistic regression model (the drag coefficient of hazelnuts).

	R^2^	MSE	MAE
Moisture	0.681	0.046	0.159
Mass	0.770	0.024	0.119
Density	0.664	0.035	0.155
Projected area	0.743	0.029	0.132
Surface area	0.814	0.015	0.097
Geometric diameter	0.863	0.012	0.081
Average	0.756	0.027	0.124

**Table 3:** Evaluation of ANN model (hazelnut terminal velocity).

	R^2^	MSE	MAE
Moisture	0.894	0.010	0.083
Mass	0.901	0.009	0.067
Density	0.882	0.013	0.078
Projected area	0.943	0.006	0.066
Surface area	0.920	0.008	0.072
Geometric diameter	0.949	0.005	0.053
Average	0.915	0.009	0.070

**Table 4:** Evaluation of ANN model (the drag coefficient of hazelnuts).

	R^2^	MSE	MAE
Moisture	0.785	0.016	0.109
Mass	0.899	0.011	0.073
Density	0.932	0.007	0.066
Projected area	0.785	0.017	0.101
Surface area	0.866	0.013	0.081
Geometric diameter	0.889	0.009	0.084
Average	0.859	0.012	0.086

**Table 5:** Evaluation of SVR model (hazelnut terminal velocity).

	R^2^	MSE	MAE
Moisture	0.849	0.140	0.105
Mass	0.858	0.013	0.103
Density	0.914	0.009	0.084
Projected area	0.850	0.015	0.118
Surface area	0.802	0.020	0.121
Geometric diameter	0.880	0.012	0.090
Average	0.859	0.035	0.104

**Table 6:** Evaluation of SVR model (hazelnut drag coefficient).

	R^2^	MSE	MAE
Moisture	0.657	0.026	0.136
Mass	0.794	0.022	0.119
Density	0.914	0.009	0.090
Projected area	0.791	0.017	0.107
Surface area	0.827	0.016	0.109
Geometric diameter	0.830	0.015	0.099
Average	0.802	0.018	0.110

**Table 7:** Comparison of terminal velocity of hazelnut estimation methods.

	LR	ANN	SVR
R^2^	0.690	0.915	0.859
MSE	0.031	0.009	0.035
MAE	0.158	0.070	0.104

**Table 8:** Comparison of drag coefficient of hazelnut estimation methods.

	LR	ANN	SVR
R^2^	0.756	0.859	0.802
MSE	0.027	0.012	0.018
MAE	0.124	0.086	0.110

## Data Availability

The data used to support the findings of this study can be made available by the corresponding author upon request.

## Supplementary Materials

The following supporting information can be downloaded at: https://www.mdpi.com/article/10.3390/foods12152879/s1, Figure S1: Linear regression scatter plot of the velocity of hazelnuts. Figure S2: Linear regression scatter plot of the drag coefficient of hazelnuts. Figure S3: ANN scatter plot of the velocity of hazelnut. Figure S4: ANN scatter plot of the drag coefficient of hazelnut. Figure S5: SVR scatter plot of the velocity of hazelnut. Figure S6: SVR scatter plot of the drag coefficient of hazelnut.
