# Supplementary material for: Nondestructive Estimation of Hazelnut (Corylus avellana L.) Terminal Velocity and Drag Coefficient Based on Some Fruit Physical Properties Using Machine Learning Algorithms
Source: Foods. 2023 Jul 28;12(15):2879. doi: 10.3390/foods12152879 (PMC10417351; doi:10.3390/foods12152879)
Supplement: Supplementary file 1 [file foods-12-02879-s001.zip › foods-2526853-supplementary.pdf]

Figure 1 shows scatter plot of the velocity of hazelnuts for logistic regression. There is a strong and positive relationship between the velocity of hazelnut and its predicted results. As one variable value increases, so does the other, with the dots clustered near the line.

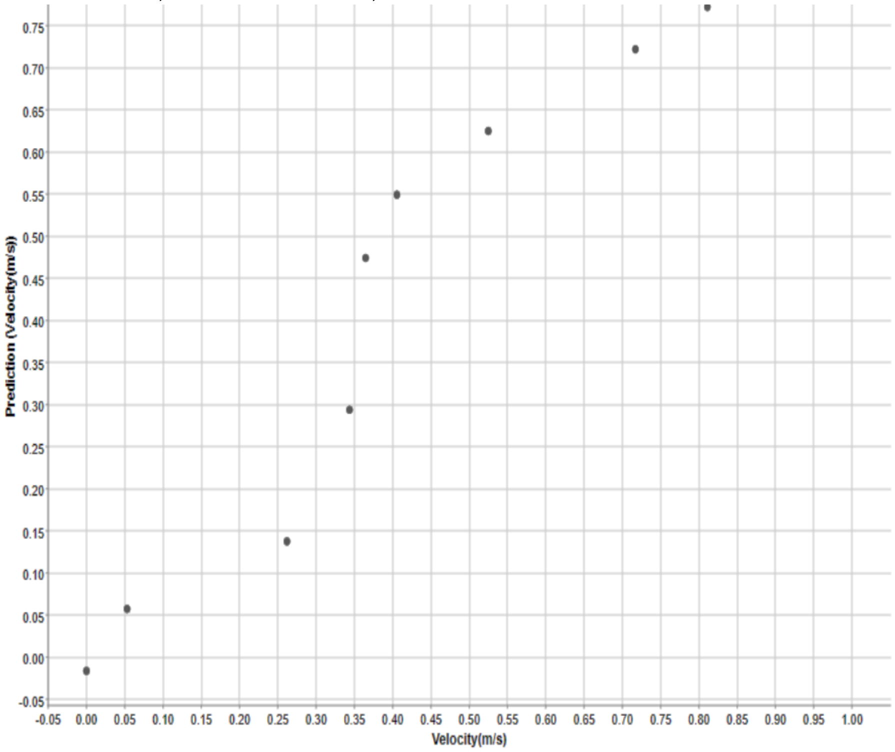

a) Moisture

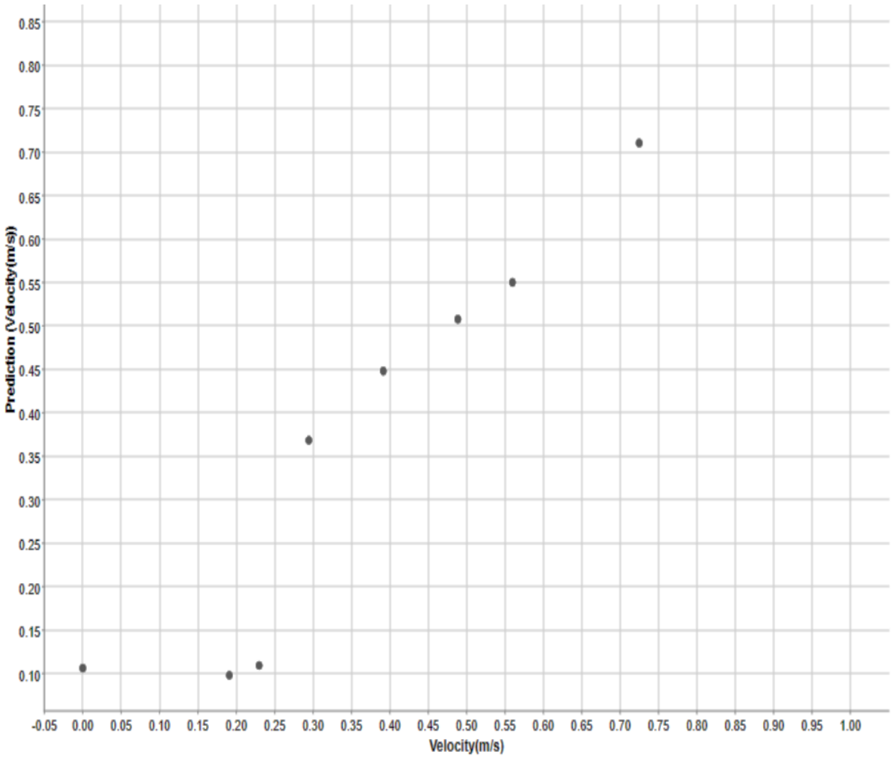

b) Mass

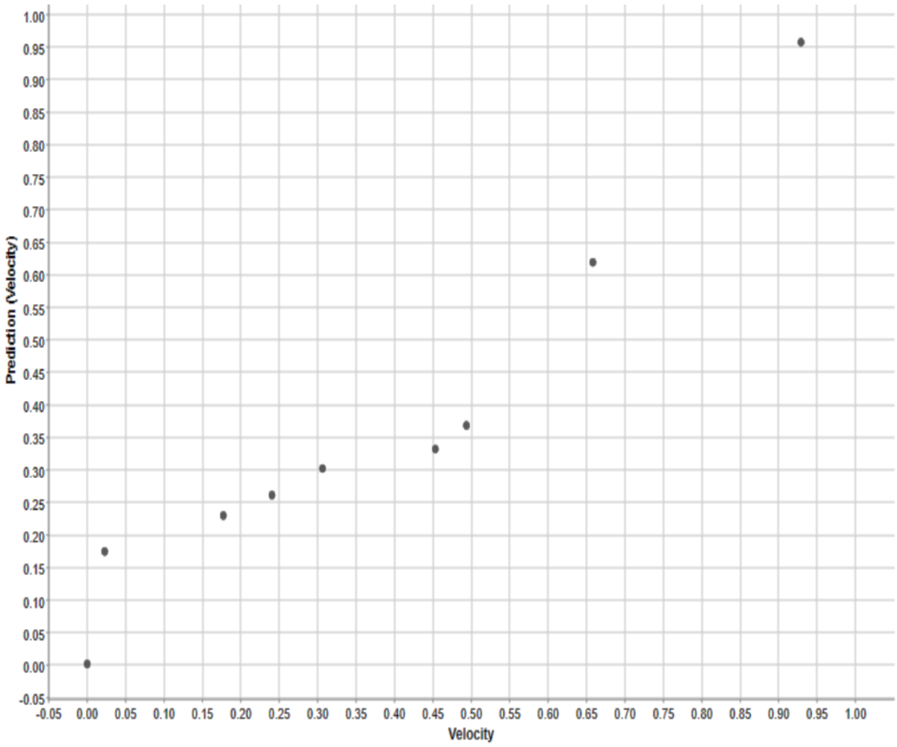

c) Density

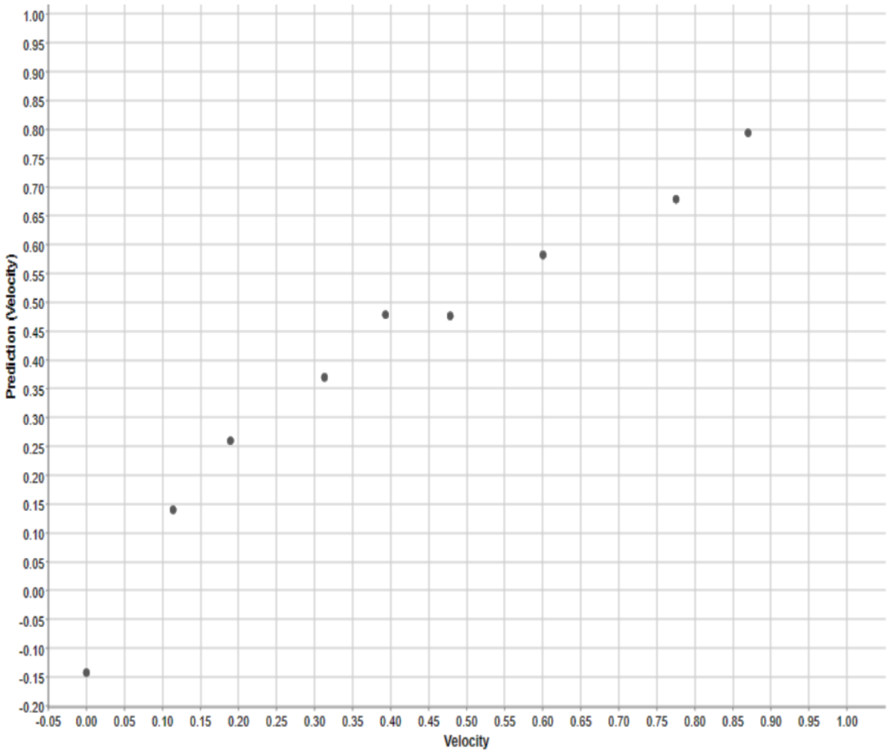

d) Projected area

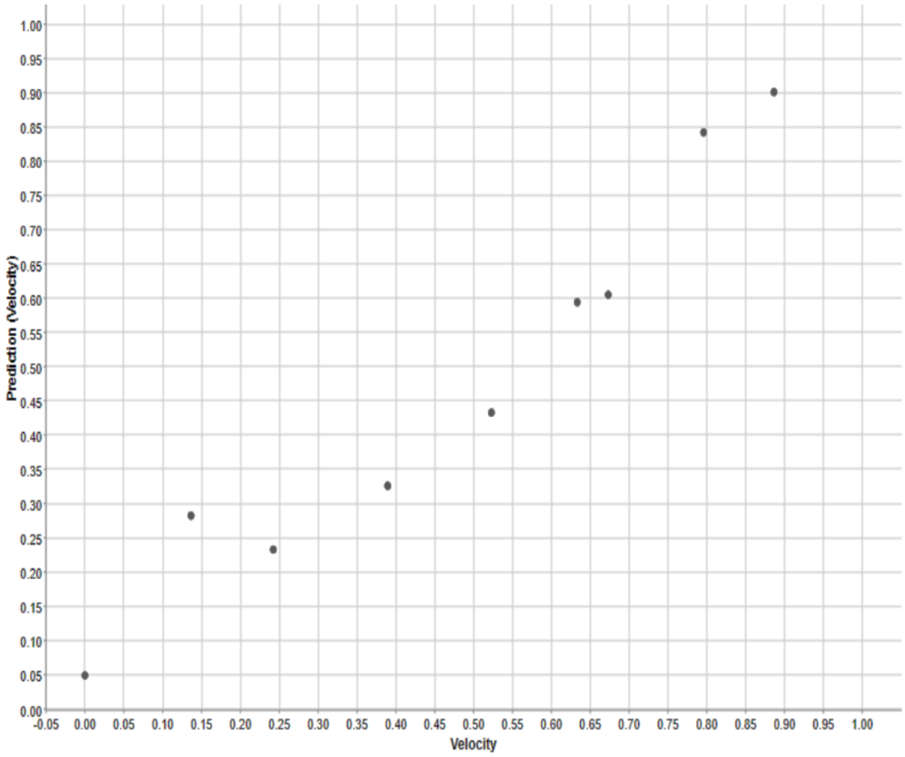

e) Surface area

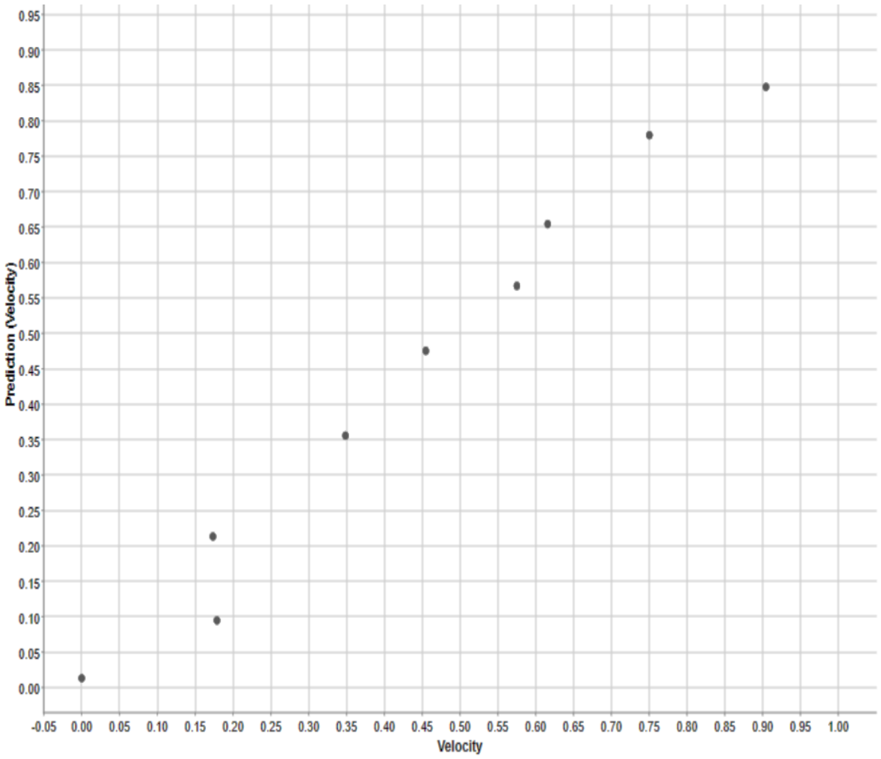

f) Geometric diameter

Figure S1. Linear regression scatter plot of the velocity of hazelnuts

Figure 2 shows scatter plot of the velocity of hazelnuts for logistic regression. There is a strong and positive relationship between the drag coefficient of hazelnut and its predicted results. As one variable value increases, so does the other, with the dots clustered near the line.

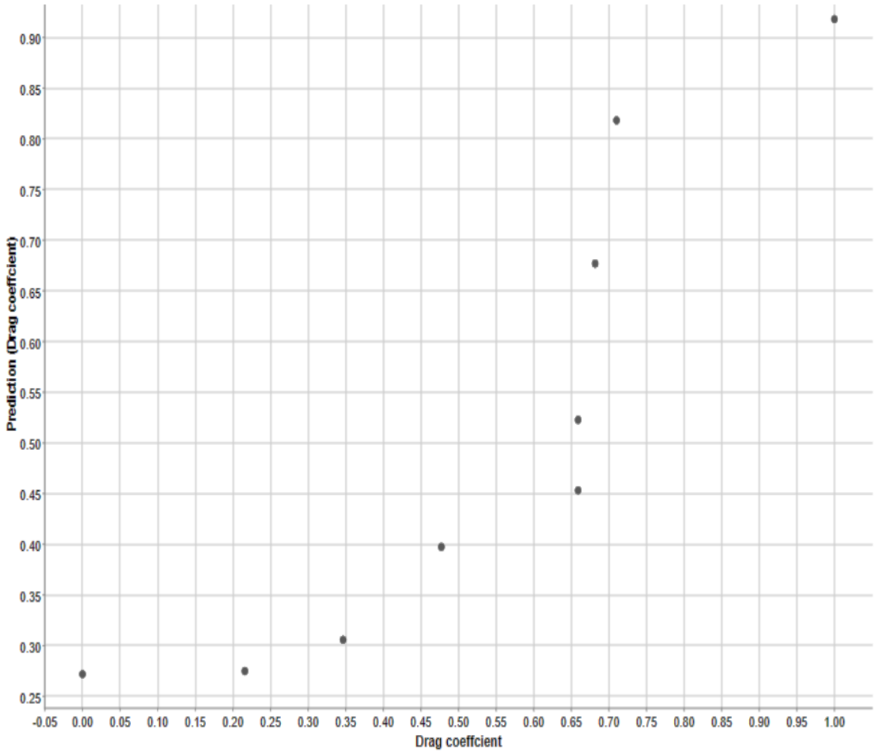

a) Moisture

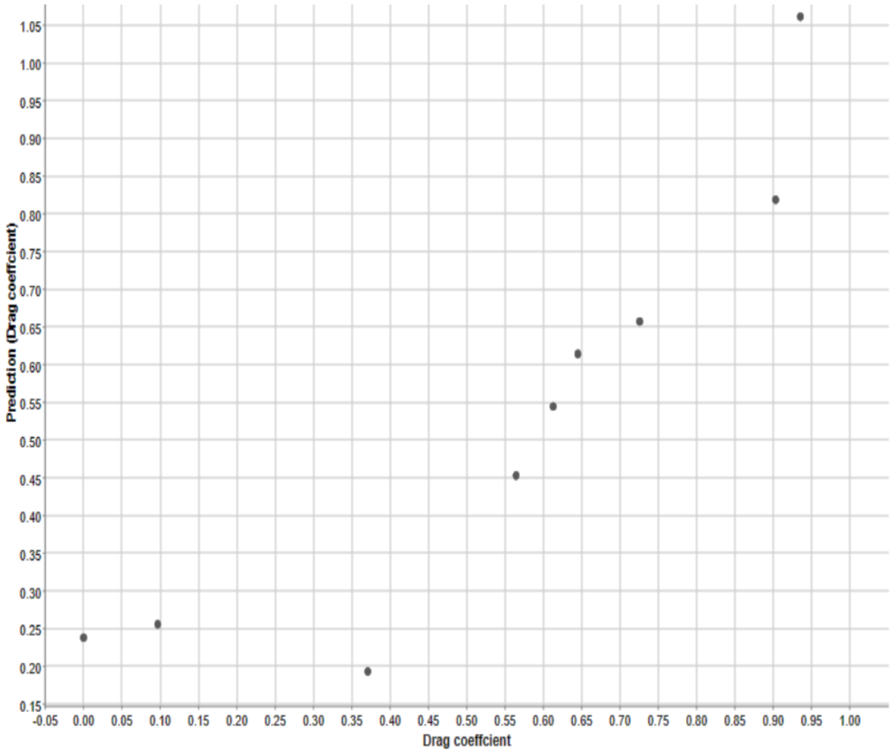

b) Mass

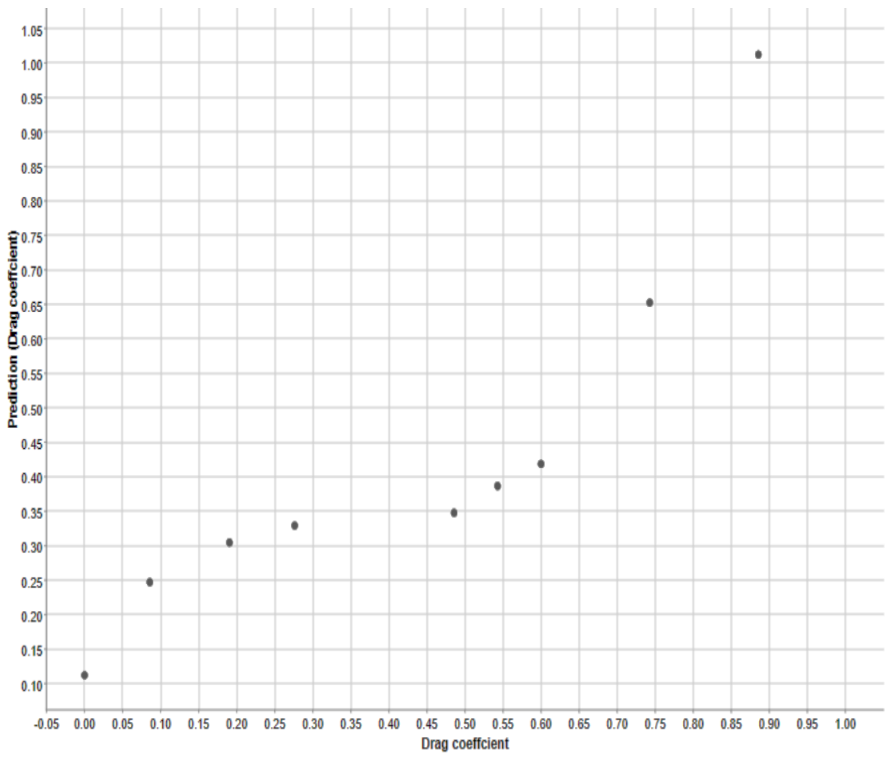

c) Density

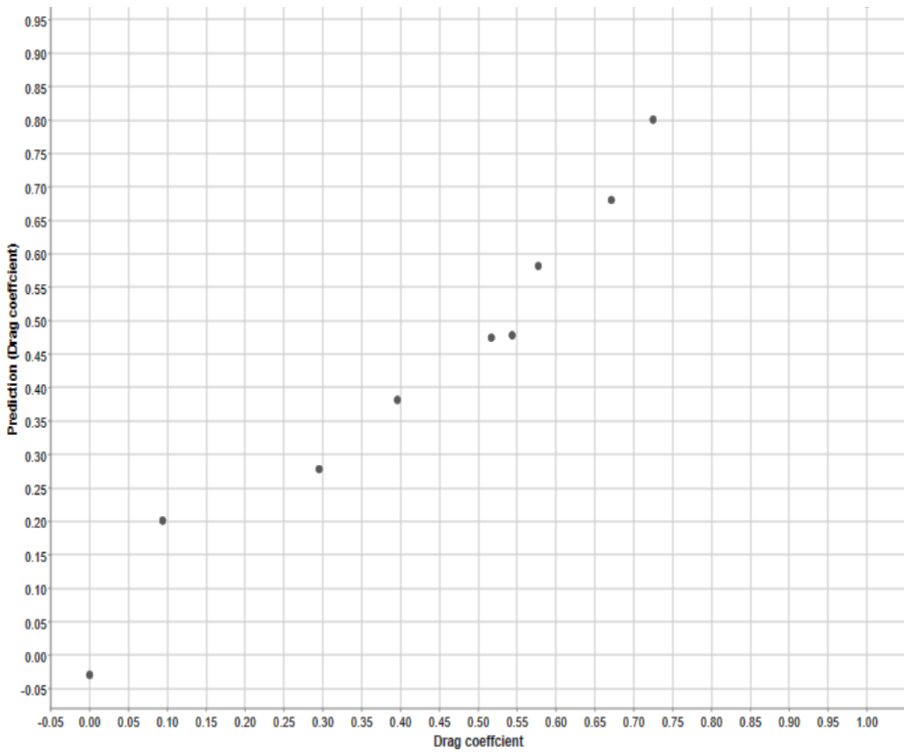

d) Projected area

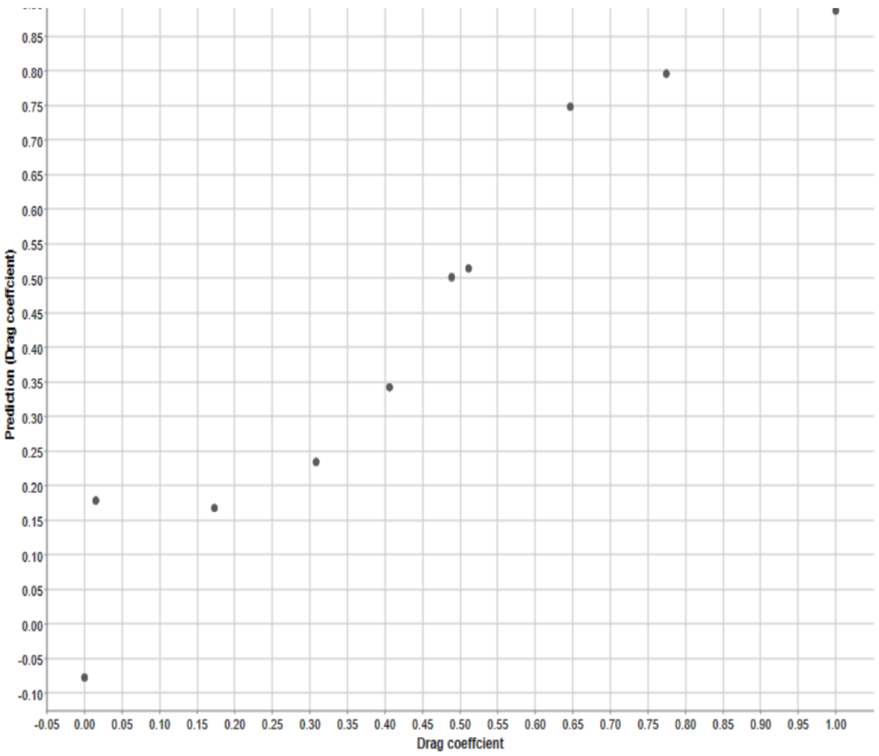

e) Surface area

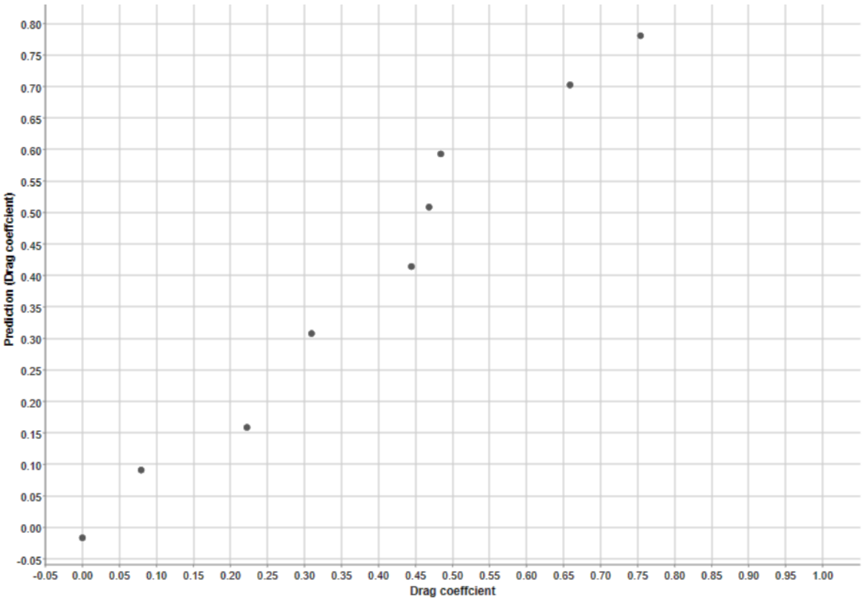

f) Geometric diameter

Figure S2. Linear regression scatter plot of the drag coefficient of hazelnuts

Figure 3 shows scatter plot of the velocity of hazelnuts for ANN. There is a strong and positive relationship between velocity of hazelnut and its predicted results. As one variable value increases, so does the other, with the dots clustered near the line.

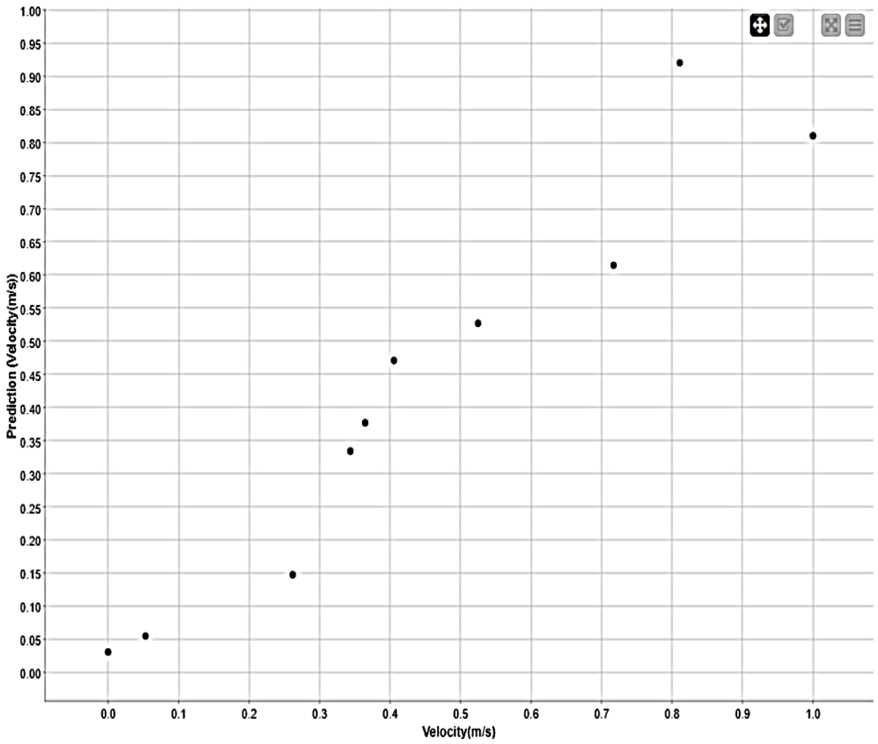

a) Moisture

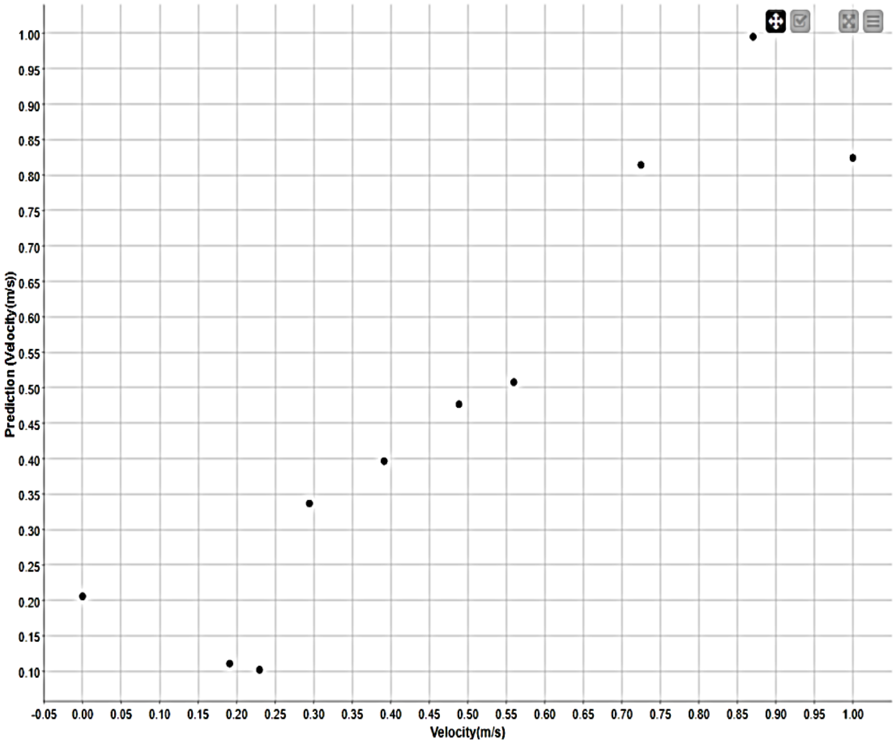

b) Mass

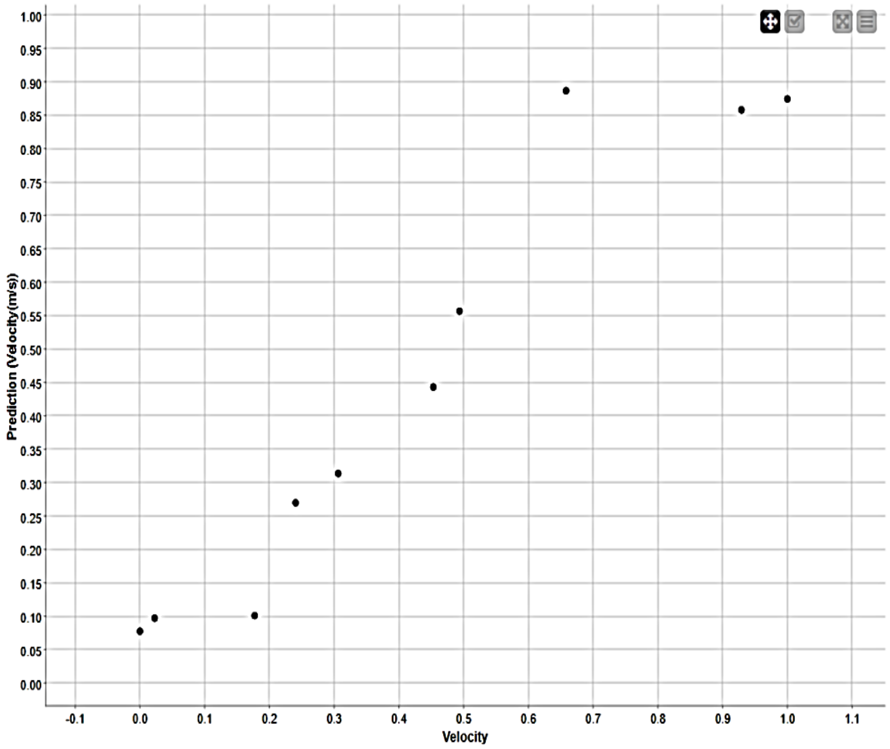

c) Density

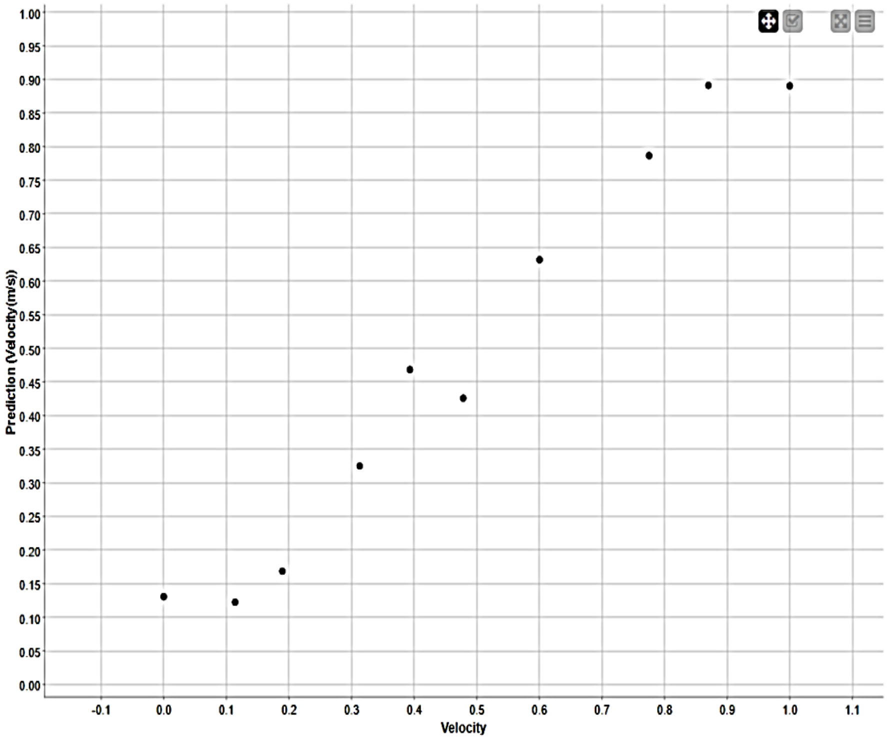

d) Projected area

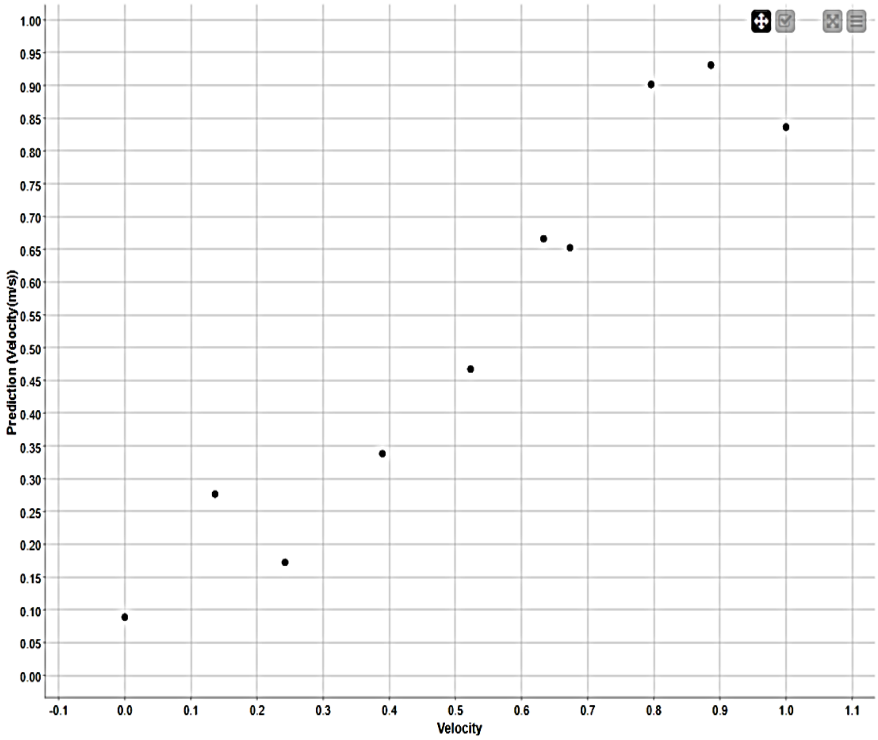

e) Surface area

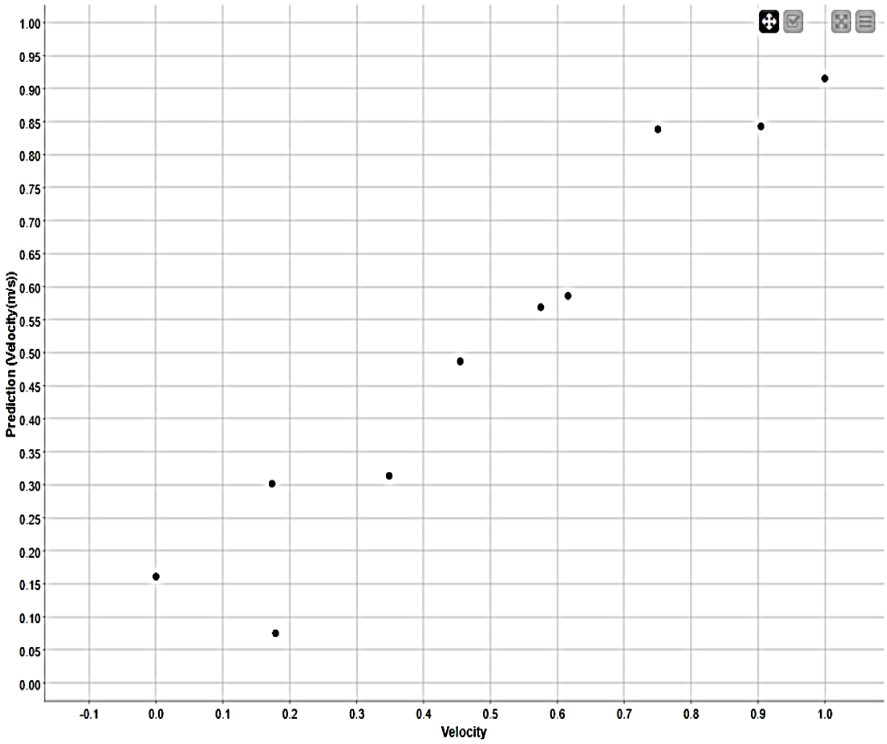

f) Geometric diameter

Figure S3. ANN scatter plot of the velocity of hazelnut.

Figure 4 shows a scatter plot of the drag coefficient of hazelnut for ANN. There is a positive and weak relationship between the drag coefficient of hazelnut and its predicted results (moisture). The value of both variables increases at the same time, but the dots are scattered. There is a positive and strong relationship between the drag coefficient of hazelnut and its predicted results. As one variable value increases, so does the other, with the dots clustered near the line.

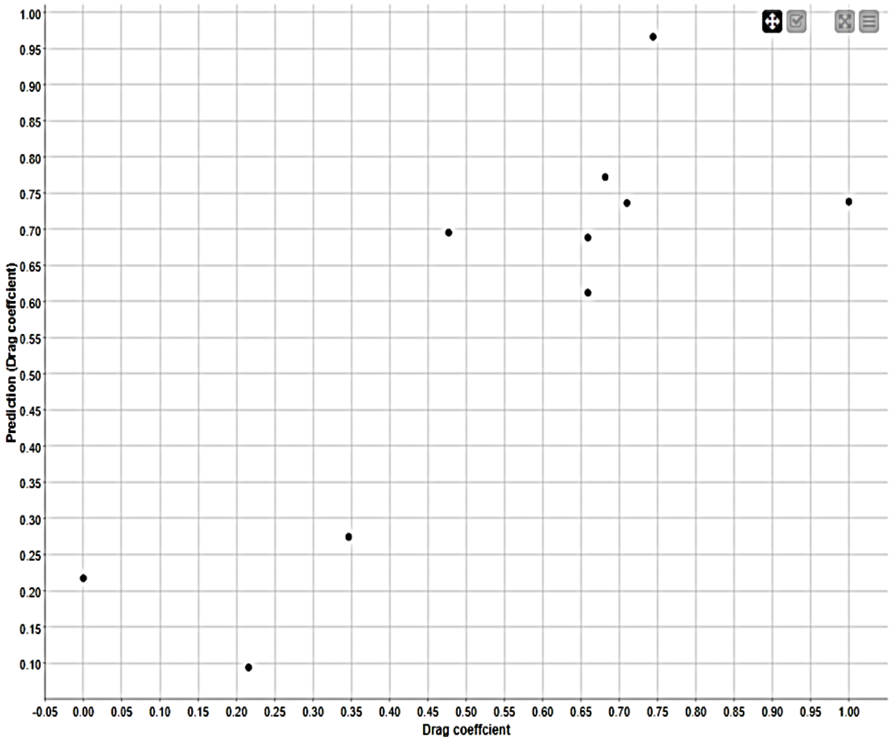

a) Moisture

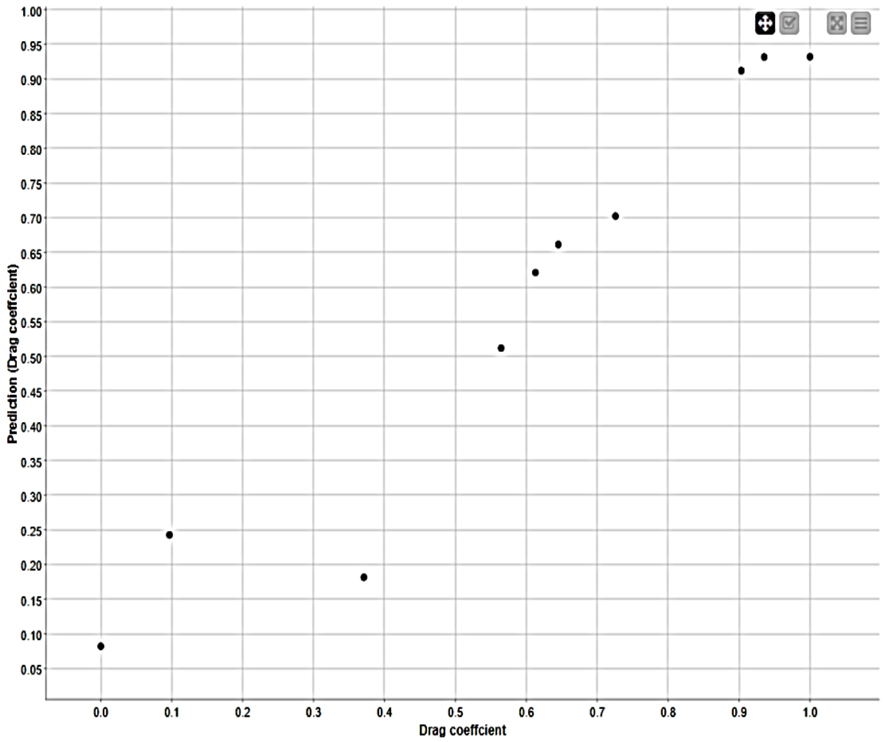

b) Mass

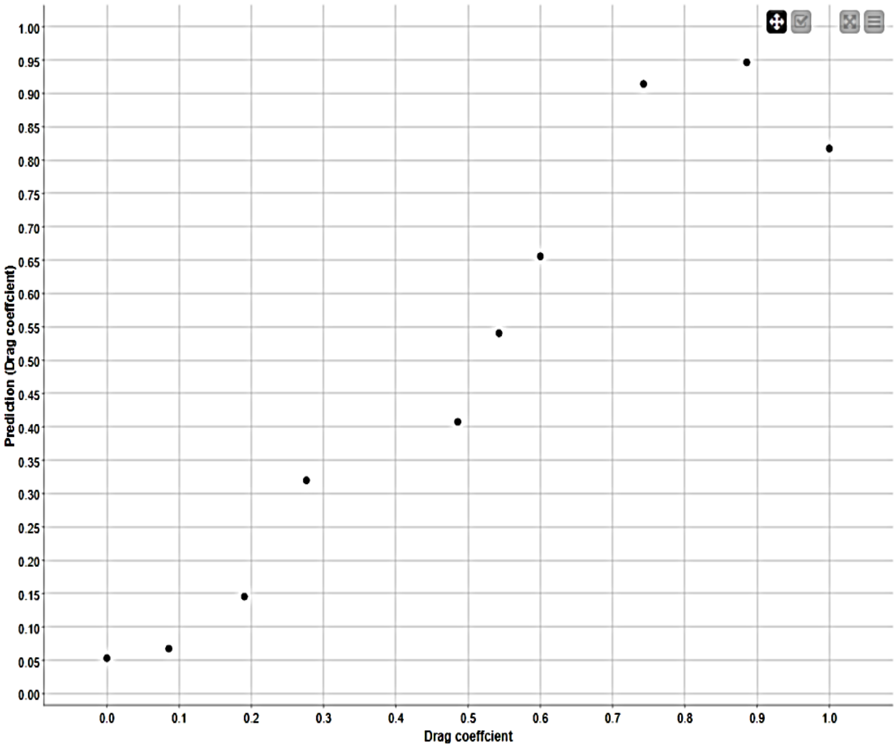

c) Density

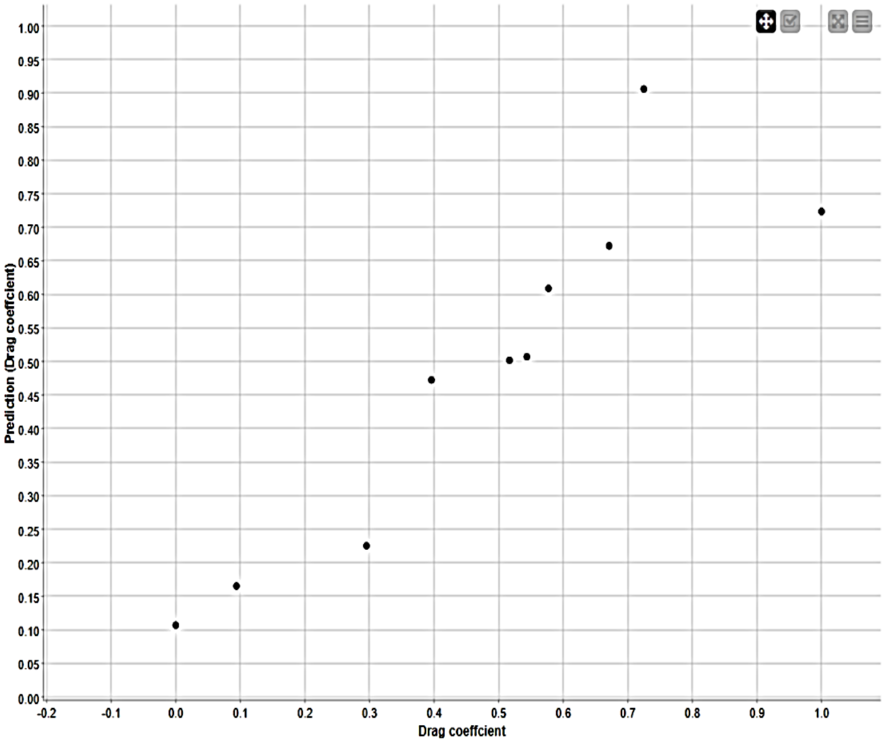

d) Projected area

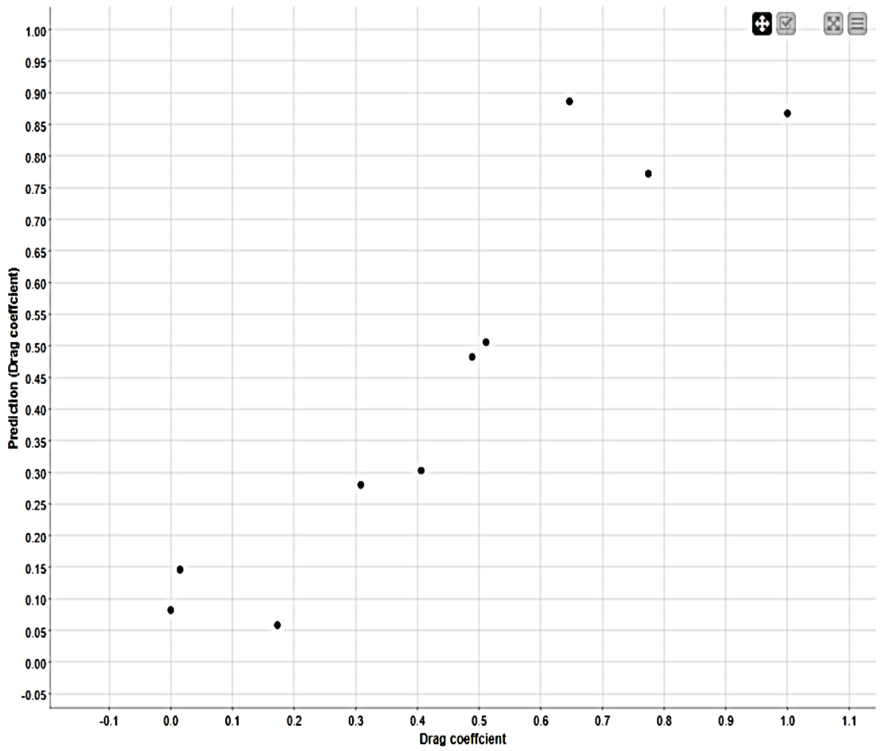

e) Surface area

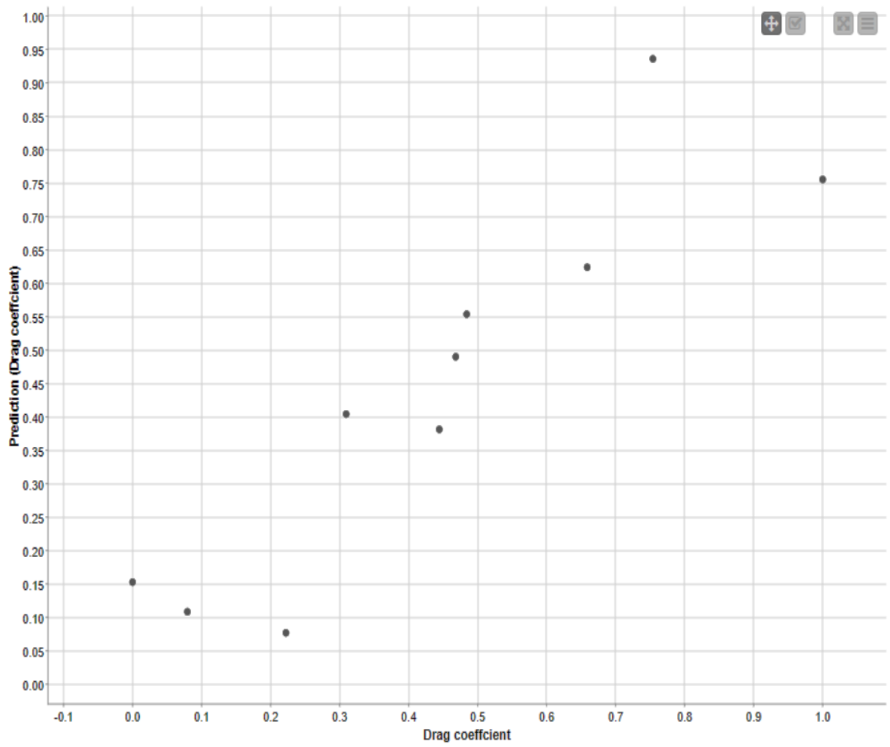

f) Geometric diameter

Figure S4. ANN scatter plot of the drag coefficient of hazelnut.

Figure 5 shows scatter plot of the velocity of hazelnuts for SVR. There is a strong and positive relationship between the velocity of hazelnut and its predicted results. As one variable value increases, so does the other, with the dots clustered near the line.

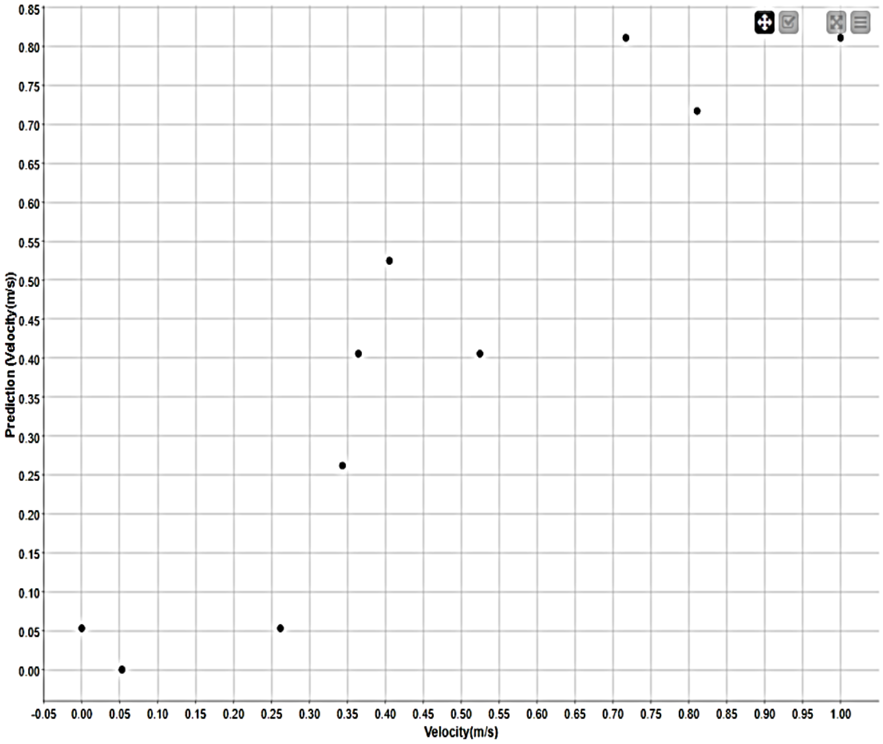

a) Moisture

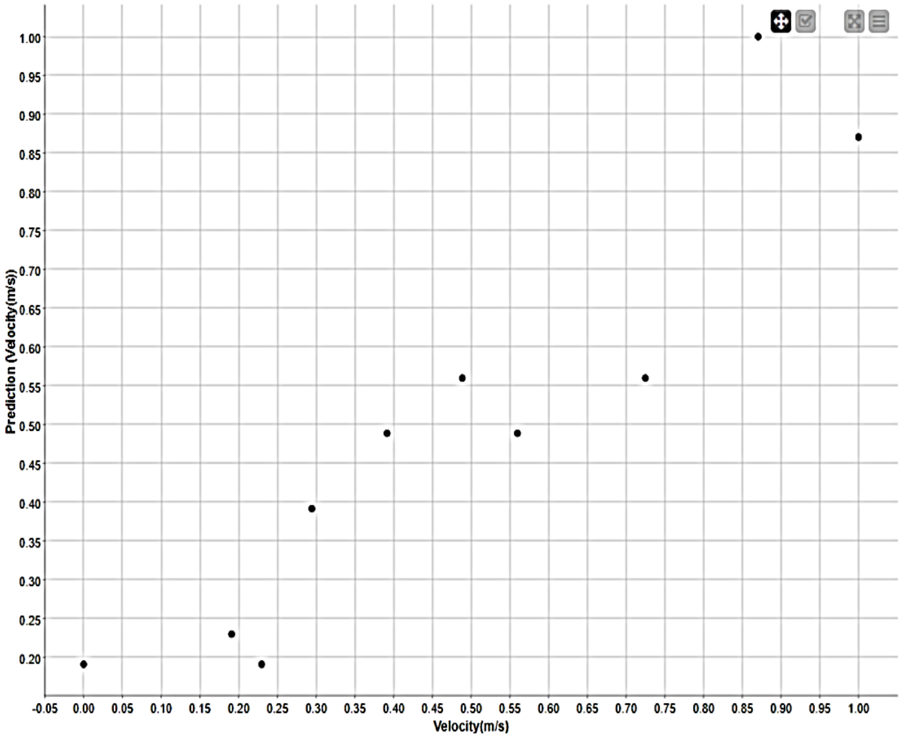

b) Mass

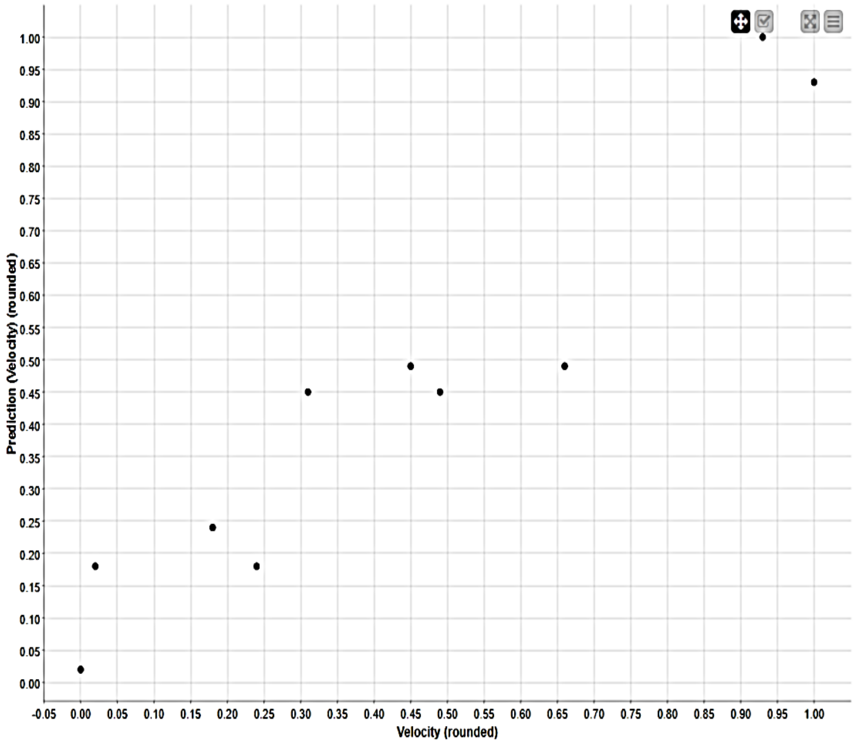

c) Density

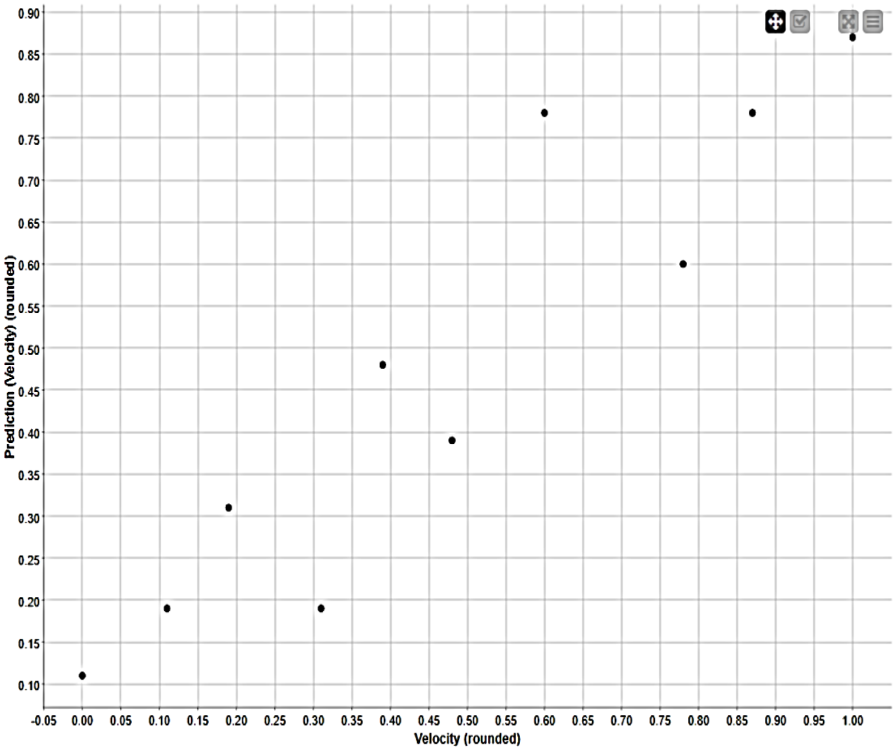

d) Projected area

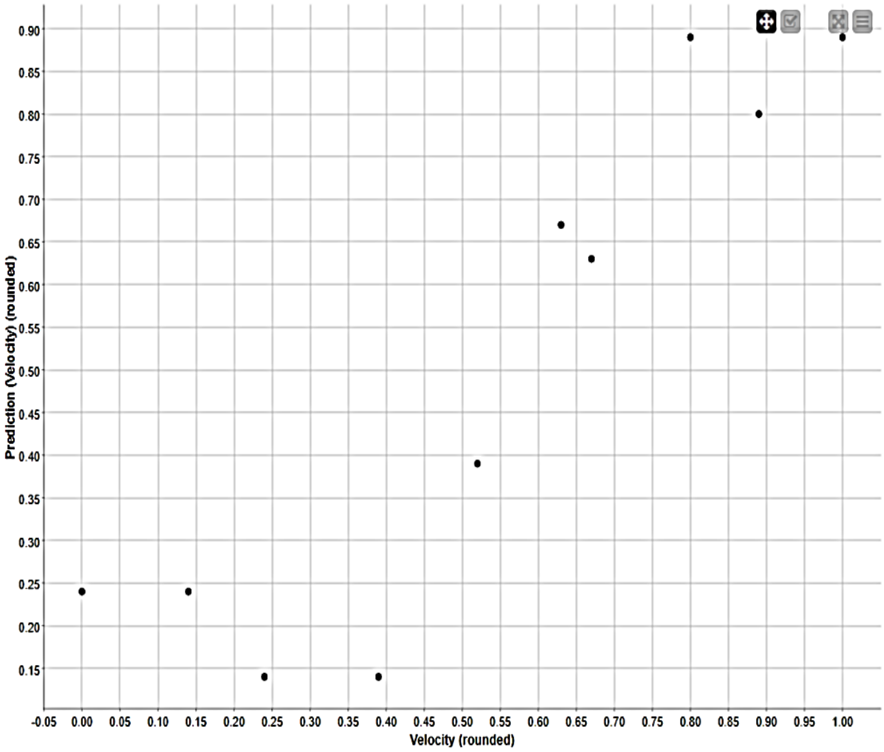

e) Surface area

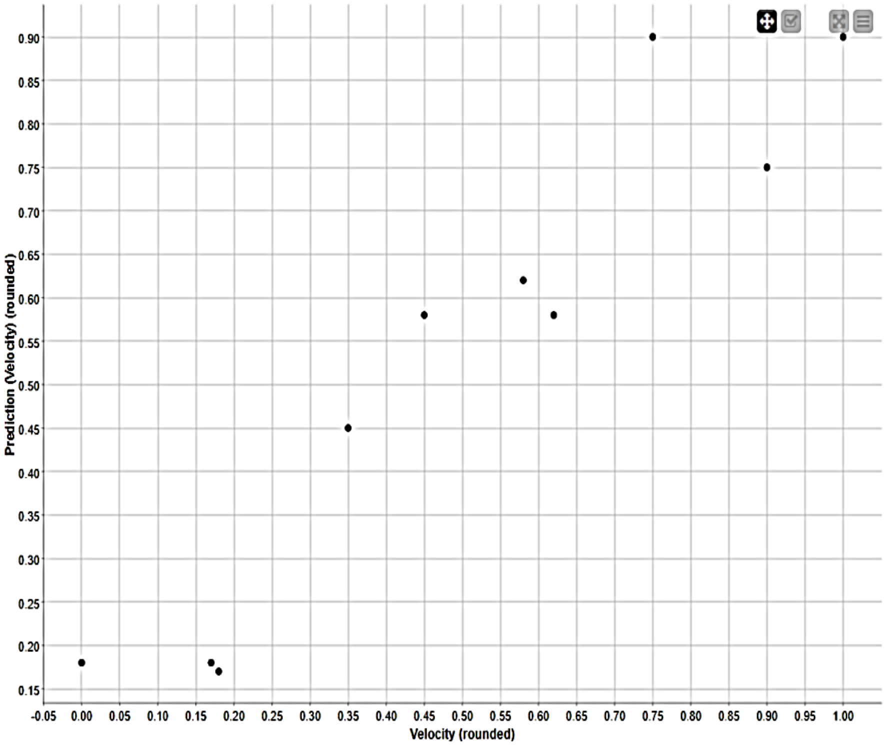

f) Geometric diameter

Figure S5. SVR scatter plot of the velocity of hazelnut

Figure 6 shows scatter plot of the drag coefficient of hazelnut for SVR. There is a strong and positive relationship between the velocity of hazelnut and its predicted results. As one variable value increases, so does the other, with the dots clustered near the line.

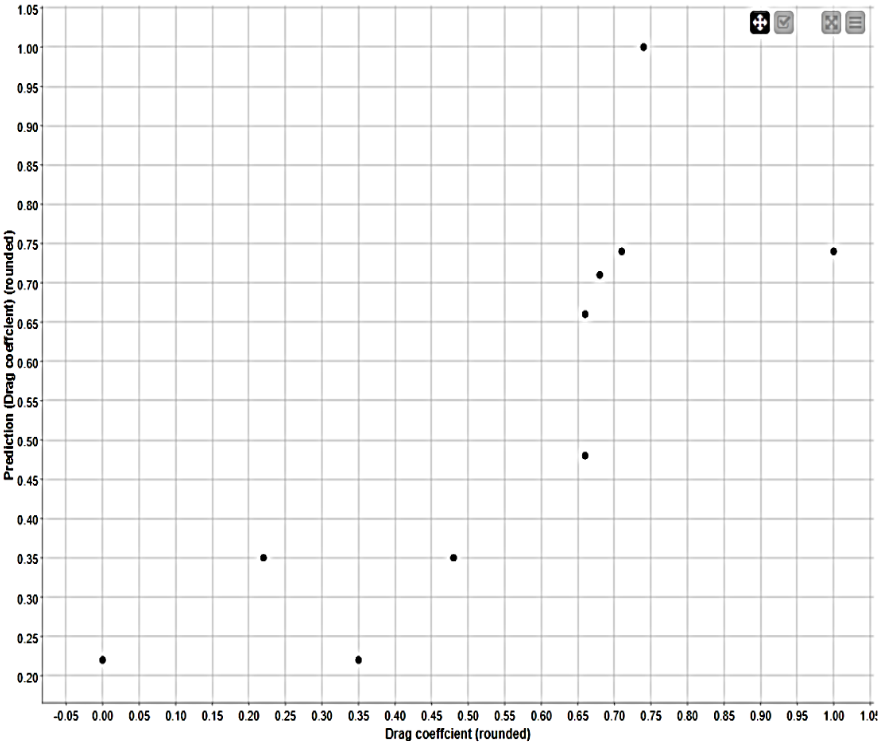

a) Moisture

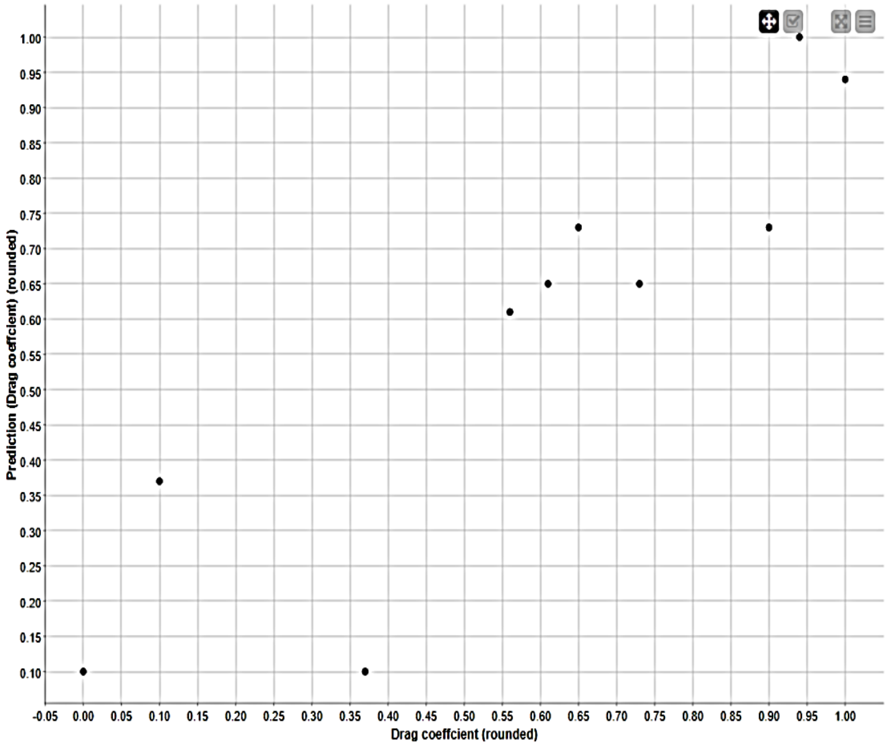

b) Mass

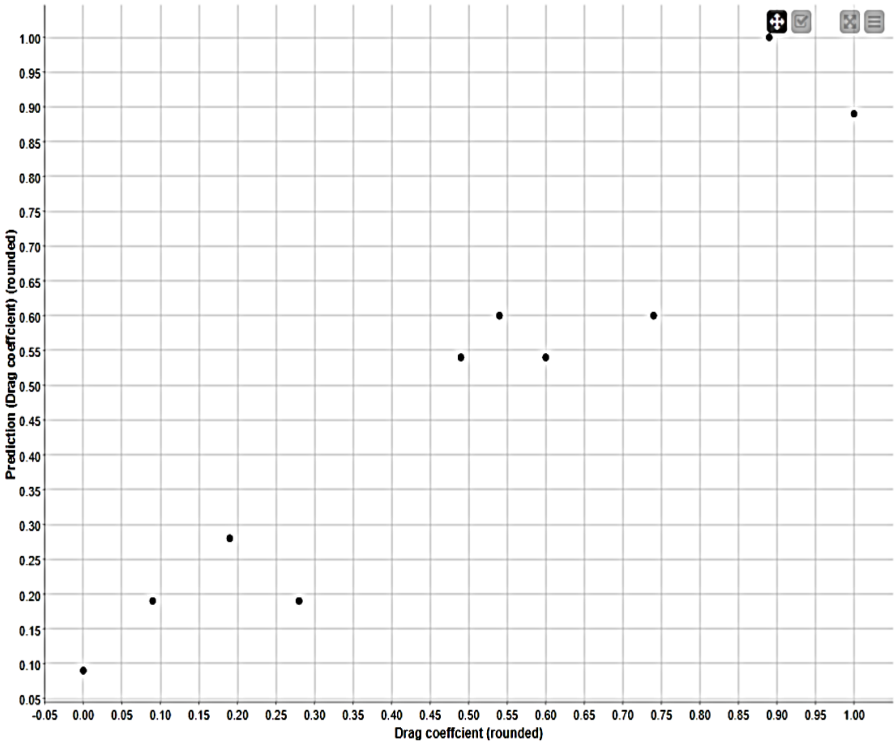

c) Density

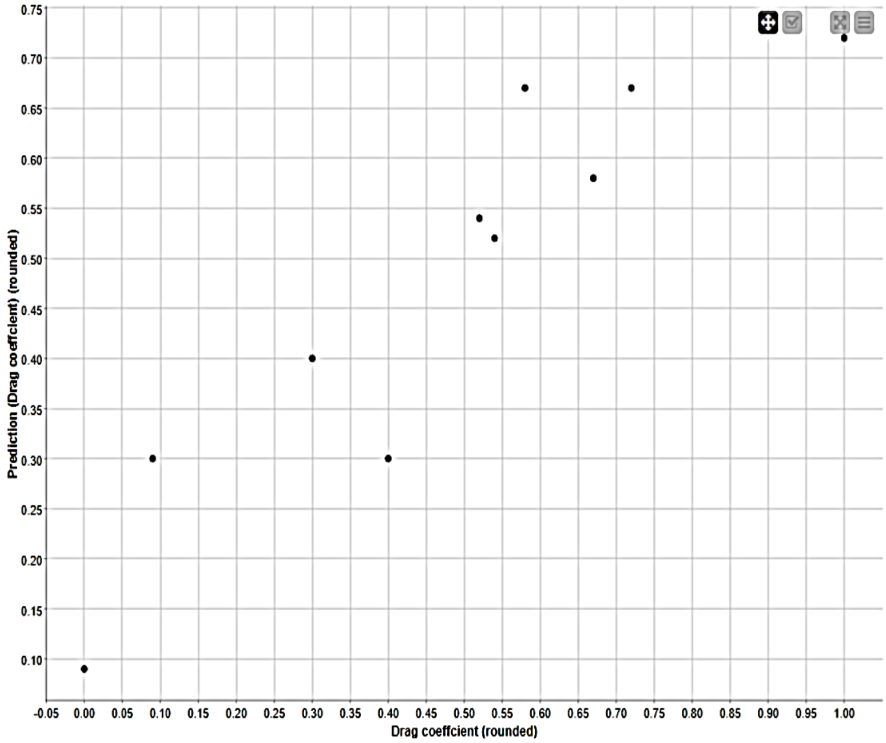

d) Projected area

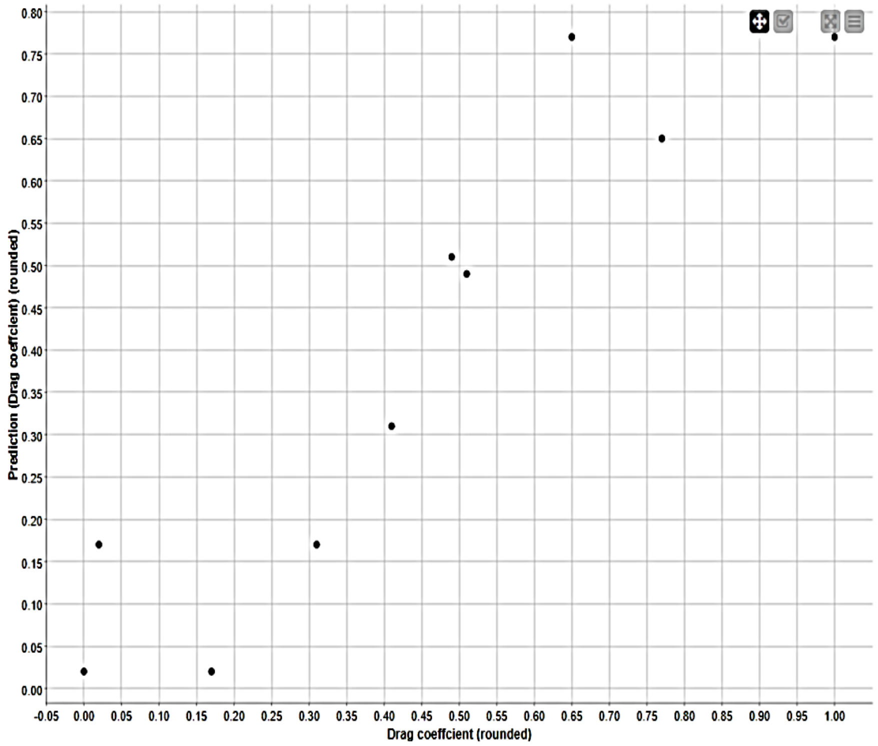

e) Surface area

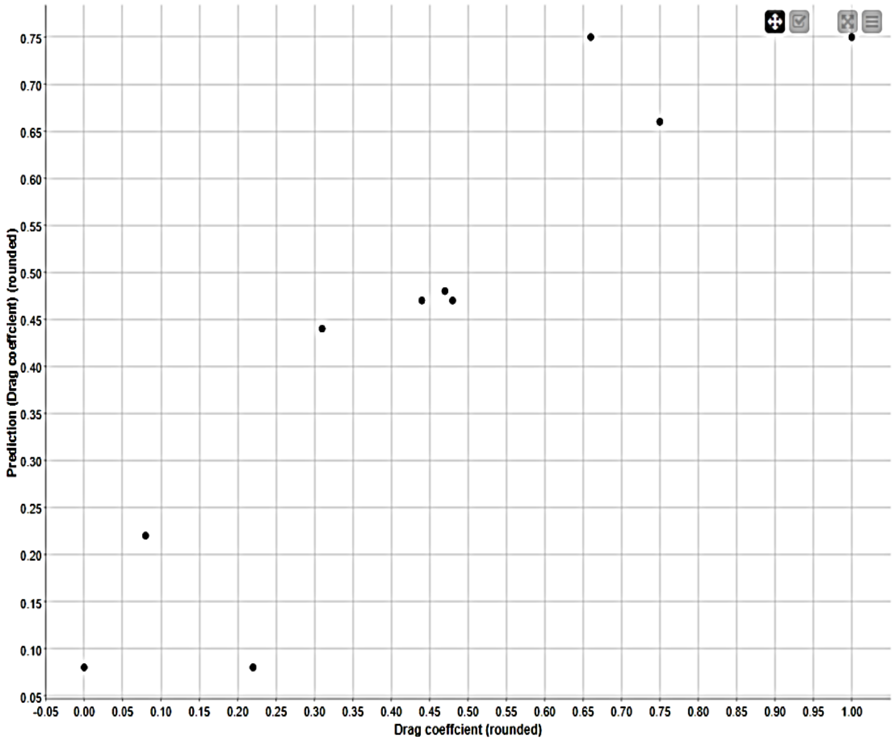

f) Geometric diameter

Figure S6. SVR scatter plot of the drag coefficient of hazelnut
